# Efficient marker free CRISPR/Cas9 genome editing for functional analysis of gene families in filamentous fungi

**DOI:** 10.1186/s40694-019-0076-7

**Published:** 2019-09-21

**Authors:** Tim M. van Leeuwe, Mark Arentshorst, Tim Ernst, Ebru Alazi, Peter J. Punt, Arthur F. J. Ram

**Affiliations:** 1 Department Molecular Microbiology and Biotechnology, Institute of Biology, Leiden University, Sylviusweg 72, 2333 BE Leiden, The Netherlands; 2 Dutch DNA Biotech, Hugo R Kruytgebouw 4-Noord, Padualaan 8, 3584 CH Utrecht, The Netherlands; 3 Present Address: Dutch DNA Biotech, Hugo R Kruytgebouw 4-Noord, Padualaan 8, 3584 CH Utrecht, The Netherlands

**Keywords:** CRISPR/Cas9, Gene editing, Marker free, Multiplexing, Knockouts, Gene families, Cell wall, *Aspergillus niger*, *pyrG* marker effects

## Abstract

**Background:**

CRISPR/Cas9 mediated genome editing has expedited the way of constructing multiple gene alterations in filamentous fungi, whereas traditional methods are time-consuming and can be of mutagenic nature. These developments allow the study of large gene families that contain putatively redundant genes, such as the seven-membered family of *crh*-genes encoding putative glucan–chitin crosslinking enzymes involved in cell wall biosynthesis.

**Results:**

Here, we present a CRISPR/Cas9 system for *Aspergillus niger* using a non-integrative plasmid, containing a selection marker, a Cas9 and a sgRNA expression cassette. Combined with selection marker free knockout repair DNA fragments, a set of the seven single knockout strains was obtained through homology directed repair (HDR) with an average efficiency of 90%. Cas9–sgRNA plasmids could effectively be cured by removing selection pressure, allowing the use of the same selection marker in successive transformations. Moreover, we show that either two or even three separate Cas9–sgRNA plasmids combined with marker-free knockout repair DNA fragments can be used in a single transformation to obtain double or triple knockouts with 89% and 38% efficiency, respectively. By employing this technique, a seven-membered *crh*-gene family knockout strain was acquired in a few rounds of transformation; three times faster than integrative selection marker (*pyrG*) recycling transformations. An additional advantage of the use of marker-free gene editing is that negative effects of selection marker gene expression are evaded, as we observed in the case of disrupting virtually silent *crh* family members.

**Conclusions:**

Our findings advocate the use of CRISPR/Cas9 to create multiple gene deletions in both a fast and reliable way, while simultaneously omitting possible locus-dependent-side-effects of poor auxotrophic marker expression.

## Introduction

The fungal cell wall is comprised of a series of different polymeric sugars, such as alpha-glucans, beta-glucans, chitin (poly-1,4-linked *N*-acetyl-glucosamine), galactomannans and mannoproteins. These structural components are synthesized by membrane localized alpha-glucan synthases (Ags-proteins), beta glucan-synthases (Fks- or Bgs-proteins), chitin synthases (Chs-proteins) or are assembled in the secretion pathway (galactomannans and mannoproteins). For an extensive review on cell wall organization and biosynthesis we refer to Free, 2013 [1]. The individual components are often cross-linked to each other by extracellular transglycosidases including the beta-glucan crosslinking enzymes (Gas or Gas1p/GEL1/Phr1p family-proteins [2–4]) or beta-glucan–chitin cross linking enzymes (Crh/Utr family-proteins [5–8]). For a comprehensive review on glucan–chitin cross-linking we refer to Arroyo et al. [9]. The interlinked cell wall matrix forms the physical barrier between the outside world and the inside of the cell, providing structural integrity and protection from biotic and abiotic factors.

Genome sequences of filamentous fungi, including *Aspergillus niger,* have shown that these cell wall related synthases and crosslinking enzymes often consist of large gene families. For example, the *A. niger* genome contains five Ags homologs, nine Chs homologs, seven Gas/GEL homologs and seven Crh-homologs [10]. The high number of genes in these families may correlate to the complexity of the multicellular, filamentous life style and offers the fungus to regulate the expression of these genes both in time and space during development or in response to stress. To perform functional analysis and determine possible redundancy of genes within a gene family, the construction of a single strain with multiple gene deletions is desirable.

Current methods to create multiple gene deletions in *A. niger* include the use of the *pyrG* or *amdS*-based transformation system combined with the subsequent recycling of *pyrG* or *amdS* via counter selection approach using 5-fluoroorotic acid (5-FOA) or 5-fluoro-acetamide (5-FAA) [11], respectively. Another approach is the use of multiple auxotrophic strains, but limits one to a total of four separate selection markers in *A. niger* (*pyrG*, *nicB*, *argB*, *adeA*) [12]. Dominant markers such as hygromycin or phleomycin resistance genes [13] can additionally be used. The recycling method is time consuming and the use of auxotrophic markers require supplementation when not all auxotrophic markers are used which can influence the growth phenotype of the strain, and both are therefore undesirable.

Alternatively, the CRISPR/Cas9 era has opened up the possibility to target and alter genes in an effective way with the potential to be selection marker free, hypothetically allowing limitless genetic alterations. For *A. niger*, several studies have been published to demonstrate the potential of CRISPR/Cas9, yet many still rely on the integration of selection marker in which the repair DNA fragment either remains integrated [14–16] or allows subsequent “pop-out” (*pyrG*) using 5-FOA counter selection [17, 18]. Additionally, some studies have reported the use of marker-free deletion of single genes in one transformation, using repair DNA fragment(s) in combination with either integrative pUC-based plasmids [19] or plasmids with self-replicating extrachromosomal AMA1 elements [20, 21]. As such, these developments have expedited the possibility to target multiple genes in a single transformation and thereby increase efficiency of strain construction. However, no studies have reported on the ability to multiplex knockouts of different genes in a single transformation without the use of integrative selection markers. To circumvent the time-consuming recycling of markers or the use of multiple auxotrophic strains to generate multiple gene deletion mutants, we demonstrate a marker-free CRISPR/Cas9-based transformation procedure. This procedure allows us to knock out multiple genes in one transformation in which the transformed strain can immediately be reused for subsequent transformations, if desired. Moreover, this marker-free approach excludes the possibility for “position effects” from genomic loci with low overall gene expression causing lack of marker gene expression [22–25]. Here, efficiency of multiplex knockout strain construction is demonstrated, using Cas9–sgRNA plasmids together with marker-free repair DNA fragments by knocking out the seven-membered cell wall chitin cross-linking gene family (*crhA*-*G*), without using an obvious selectable phenotype.

## Results

### CRISPR/Cas9 plasmid design and proof of functionality

Our CRISPR/Cas9 procedure combines Cas9 expression driven by the constitutive *Aspergillus nidulans tef1*-promoter from an autonomously replicating plasmid (pFC332; [32]) with expression of the sgRNA driven by the tRNA^Pro1^ RNAIII polymerase promoter [20]. The sgRNA expression cassette was amplified with extended primers to include *Pac*I restriction sites on both ends of the cassette. A unique *Pac*I restriction site in the pFC-series plasmids (pFC330-333) was used for ligation the sgRNA expression cassette into the vector (see “Methods”).

To show functionality of the CRISPR/Cas9 system, we chose to target the *brnA* gene (NRRL3_01040; An14g05370) of *A. niger* which is homologous to *Aspergillus fumigatus abr1* and encodes a multi copper oxidase that is involved in the synthesis of melanin [26, 27]. A knockout of *brnA* results in the formation of brown-colored spores, thus providing a direct read-out on transformation plates. A sgRNA target sequence for *brnA* was designed with CHOPCHOP (see “Methods”). Using fusion PCR, the *brnA*-target was cloned into a sgRNA expressing PCR fragment and ligated into pFC332 to yield pFC332_*brnA*-sgRNA (Table 1).

**Table 1:** All plasmids used in this study

Plasmid name (in text)	Technical name	Parental plasmid	Gene	An# (gene)	Gene name	Target sequence	Reference
pFC332	pFC332	–	–	–	–	–	[32]
pFC332_*brnA*-sgRNA	pTLL40.9	pFC332	NRRL3_01040	An14g05370	*brnA*	GGAGTGGTACCAATATGTGC	This study
pFC332_*crhA*-sgRNA	pTLL48.1	pFC332	NRRL3_10021	An11g01540	*crhA*	GGAGCTACCCATAATGATCC	This study
pFC332_*crhB*-sgRNA	pTLL58.1	pFC332	NRRL3_04809	An07g07530	*crhB*	GTAGGTCTTGCTCTCACACA	This study
pFC332_*crhC*-sgRNA	∆*crhC*-plasmid	pFC332	NRRL3_04315	An07g01160	*crhC*	GCTGTCGGTGCTGCAAGTCG	This study
pFC332_*crhD*-sgRNA	pTLL51.2	pFC332	NRRL3_02532	An01g11010	*crhD*	GACTGCTGTTGCGTTGGCTG	This study
pFC332_*crhE*-sgRNA	pTLL52.1	pFC332	NRRL3_01365	An13g02510	*crhE*	GCTCGTCTTGGCGTGATAGA	This study
pFC332_*crhF*-sgRNA	pTLL53.3	pFC332	NRRL3_07085	An16g02850	*crhF*	GTAACGACACATCTTTCGAC	This study
pFC332_*crhG*-sgRNA	pTLL60.1	pFC332	NRRL3_03998	An15g05350	*crhG*	GGTGTTGAGGGGGTTGCAAT	This study

To test whether both *cas9* and the *brnA*-sgRNA were expressed (and able to target the *brnA* locus), a series of transformations were performed in a non-homologous-end-joining (NHEJ) deficient strain MA234.1 (∆*kusA*) (Table 2). The ∆*kusA* (∆*ku70* orthologue) mutant cannot repair a Cas9-induced double strand break (DSB) through NHEJ and thus relies on homology directed repair (HDR). As shown in Additional file 1: Figure S1A, the transformation of MA234.1 with pFC332_*brnA*-sgRNA did not yield in any viable transformants, whereas transformation with the control plasmid, lacking a sgRNA (pFC332, Additional file 1: Figure S1B), resulted in 100 viable, black transformants, indicating that the *brnA*-sgRNA generates a DSB which cannot be repaired leading to non-viable cells in a ∆*kusA* background. Co-transformation of homology-containing knockout-repair DNA fragment consisting of fused 5′- and 3′-flanks of the *brnA* gene, together with pFC332_*brnA*-sgRNA yielded 17/17 brown transformants on the transformation plates (Additional file 1: Figure S1C). Brown spores were visible across the entire colony for all transformants, suggesting that no sectors remained untransformed. Hence, no heterokaryotic colonies were observed on the initial transformation plate or found after single streaking. Single streaking of knockout-repair DNA fragment containing transformants on MM plates containing hygromycin showed brown coloration more clearly (Additional file 1: Figure S1E). In addition, a control co-transformation with plasmid pFC332 and knockout-repair DNA fragment did not yield brown colonies (0/63, Additional file 1: Figure S1D), showing that repair DNA fragment does not integrate autonomously at the site of homology without the assistance of a Cas9–sgRNA targeting plasmid (Single streak shown in Additional file 1: Figure S1F for clarity of coloration).

**Table 2:** All strains used in this study

Name	Genotype	References
N402	*cspA1, amdS*-	[28]
MA234.1	*cspA1, ΔkusA::DR*-*amdS*-*DR*	[31]
MA169.4	*cspA1, ΔkusA::DR*-*amdS*-*DR, pyrG*-	[42]
TLF57	*cspA1, ΔkusA::DR*-*amdS*-*DR, ΔcrhA*	This study
TLF58	*cspA1, ΔkusA::DR*-*amdS*-*DR, ΔcrhB*	This study
TLF59	*cspA1, ΔkusA::DR*-*amdS*-*DR, ΔcrhC*	This study
TLF60	*cspA1, ΔkusA::DR*-*amdS*-*DR, ΔcrhD*	This study
TLF61	*cspA1, ΔkusA::DR*-*amdS*-*DR, ΔcrhE*	This study
TLF62	*cspA1, ΔkusA::DR*-*amdS*-*DR, ΔcrhF*	This study
TLF63	*cspA1, ΔkusA::DR*-*amdS*-*DR, ΔcrhG*	This study
TLF65	*cspA1, ΔkusA::DR*-*amdS*-*DR, ΔcrhDEF*	This study
TLF66	*cspA1, ΔkusA::DR*-*amdS*-*DR, ΔcrhABDEF*	This study
TLF67	*cspA1, ΔkusA::DR*-*amdS*-*DR, ΔcrhADEFG*	This study
TLF68	*cspA1, ΔkusA::DR*-*amdS*-*DR, ΔcrhABDEFG*	This study
TLF39	*cspA1, ΔkusA::DR*-*amdS*-*DR, ΔcrhABCDEFG*	This study
MA628.1	*cspA1, ΔkusA::DR*-*amdS*-*DR, pyrG*-*, ΔcrhA::DR*-*AOpyrG*-*DR*	This study
MA629.1	*cspA1, ΔkusA::DR*-*amdS*-*DR, pyrG*-*, ΔcrhB::DR*-*AOpyrG*-*DR*	This study
MA630.2	*cspA1, ΔkusA::DR*-*amdS*-*DR, pyrG*-*, ΔcrhC::DR*-*AOpyrG*-*DR*	This study
MA631.2	*cspA1, ΔkusA::DR*-*amdS*-*DR, pyrG*-*, ΔcrhD::DR*-*AOpyrG*-*DR*	This study
MA632.2	*cspA1, ΔkusA::DR*-*amdS*-*DR, pyrG*-*, ΔcrhE::DR*-*AOpyrG*-*DR*	This study
MA633.2	*cspA1, ΔkusA::DR*-*amdS*-*DR, pyrG*-*, ΔcrhF::DR*-*AOpyrG*-*DR*	This study
MA634.3	*cspA1, ΔkusA::DR*-*amdS*-*DR, pyrG*-*, ΔcrhG::AOpyrG*	This study

Both brown transformants (pFC332_*brnA*-sgRNA + knockout-repair DNA fragment) and black transformants (pFC332 + repair DNA fragment) were single streaked on MM without hygromycin as described in “Methods” to assess whether the transformants could lose the plasmid without selection pressure. About 80% of the transformants were confirmed to lose their plasmid (data not shown).

Genomic DNA (gDNA) of successfully plasmid-cured transformants was isolated to genotype the *brnA* locus via a diagnostic PCR. A primer set was designed that prime outside of the repair DNA fragment (Additional file 2: Figure S2A). Amplification on gDNA of the wild type strain is expected to generate a PCR fragment of 4012 bp whereas the seamless ORF removal in the mutants is expected to generate a PCR fragment of 2411 bp. Additional file 2: Figure S2B shows the result of the diagnostic PCR. All brown transformants show a smaller PCR product at the expected size compared to both wild type strain and black transformants (pFC332 + knockout-repair DNA). Therefore, we conclude that our combined system of the pFC332 plasmid and a Pro^1^-promoter expression driven sgRNA construct are efficient, and can be successfully used in future gene editing.

### Multiplexing *crh* gene knockouts in successive transformation cycles

To assess the use of the multiplex CRISPR/Cas9 approach, we selected the seven-membered gene *crh* family. As shown in Table 3, this gene family consists of genes with a largely different expression levels, including both significantly expressed genes (e.g. *crhB, crhC* and *crhD*) and genes with very low expression levels (i.e. *crhG*) in different zones an *A. niger* colony under plate growth conditions grown on xylose [29]. Additionally, we looked into expression data of liquid fermentation conditions on both xylose and glucose to find that the expression of *crh* genes is independent of the carbon source. Also, under these growth conditions the expression of *crhG* was consistently low [30]. Expression of *crhA* and *crhC* also shows to be lower than during plate growth, whereas *crhD* is overall most highly expressed, followed by *crhB* and *crhF* (data not shown).

**Table 3:** *A. niger* colony expression levels of *crh* genes in different zones grown on xylose

	*crhA*	*crhB*	*crhC*	*crhD*	*crhE*	*crhF*	*crhG*
N402 zone 1	3.49	6.18	19.90	7.12	5.20	4.99	0.85
N402 zone 3	3.18	8.50	19.23	7.46	4.59	7.03	0.93
N402 zone 5	2.39	39.65	8.63	10.84	2.11	11.50	1.25

Seven-day old sandwiched colonies grown on xylose were used for RNA isolation and subsequent microarray analysis [29]. Distinct zones of the mycelium (zone 1, 3, and 5) harvested form the colony. Zone 1 represents the oldest or central part of the colony. Zone 5 represents the youngest or peripheral part of the colony, whereas zone 3 represents the intermediate zone. Expression levels are represented as percentage of actin expression

To target the individual *crh* genes, we constructed seven Cas9–sgRNA plasmids (pFC332_*crhA*-*G*-sgRNA). Targets of each *crh* gene were designed using CHOPCHOP and listed in Table 1. *Crh* targeting plasmids were co-transformed with PCR-amplified repair DNA fragments that consisted of a fused 5′ flank upstream of the *crh* ORF with a 3′ flank downstream of the *crh* ORF, similar as described for *brnA*. All transformations were performed in a ∆*kusA* background (MA234.1, Table 2).

Plasmid and repair DNA fragment combinations were transformed successfully to create single gene knockout mutants. Transformants were purified and cured of their plasmids through single streaks (see “Methods”). Knockouts were confirmed using diagnostic PCR, validating the functionality of all seven plasmids (Additional file 3: Figure S3). Out of thirty transformants tested, we observed twenty-seven with the correctly deleted gene; resulting in a frequency of repair DNA integration of 90% across seven independent transformations (Table 4). Transformation with plasmids pFC332_*crhC*-sgRNA, pFC332_*crhD*-sgRNA and pFC332_*crhE*-sgRNA and respective knockout-repair DNA fragments were both found to have a single tested transformant that did not display the deleted ORF (Table 4, Additional file 3: Figure S3). These transformants were likely able to evade the Cas9–sgRNA complex mediated DSBs, and were not investigated any further.

**Table 4:** Gene knockout efficiency per target in *Aspergillus niger*

Gene target	Knockout efficiency (%)^a^	∆*kusA* protoplast survival (%) knockout repair DNA fragment (with/without)
*brnA*	100% (9/9)	0% (17/0)
*crhA*	100% (5/5)	6.9% (72/5)
*crhB*	100% (5/5)	0% (12/0)
*crhC*	83% (5/6)	82% (56/46)
*crhD*	66.7% (2/3)	0% (6/0)
*crhE*	80% (4/5)	87.5% (280/245)
*crhF*	100% (2/2)	0% (2/0)
*crhG*	100% (4/4)	0% (18/0)

^a^Knockout efficiency among transformants checked by diagnostic PCR

In addition, control sgRNA-plasmid transformations of the ∆*kusA* background without knockout-repair DNA fragments did not yield transformants for *crhB*, *crhD*, *crhF* and *chrG*. The *crhA* sgRNA-plasmid transformation showed 90% fewer transformants without knockout-repair DNA fragment compared to when the knockout-repair DNA fragment was added (data not shown). In only two cases (*crhC* and *crhE*), close to equal numbers (Table 4) of transformants were observed, either with or without knockout-repair DNA fragments, indicating that these guides were less efficient in creating DSBs. This type of control sgRNA-plasmid transformation without repair DNA fragment provides a good control for sgRNA functionality, and may help to predict the knockout efficiency among transformants.

In order to examine whether multiple genes could be deleted in a single transformation, we transformed the cured ∆*crhE* strain (TLF61) with pFC332_*crhD*-sgRNA and pFC332_*crhF*-sgRNA (Table 1). Plasmids were transformed together with both knockout-repair DNA fragments for *crhD* and *crhF* and yielded eight colonies. Control transformation without any knockout-repair DNA fragment showed no colonies (data not shown). All eight transformants were plasmid-cured and were tested for the knockout of both *crhD* and *crhF* by diagnostic PCR. Six out of the eight transformants were found to have a double deletion of both *crhD* and *crhF* (Additional file 3: Figure S3). Subsequently, we continued using ∆*crhDEF* (TLF65) for a successive round of transformation.

Inspired by the successful transformation with two separate plasmids harboring the same hygromycin selection marker, we decided to perform both additional double and also triple gene targeting transformations. To construct quintuple mutants *(*∆*crhABDEF)* and *(*∆*crhADEFG)* or sextuple mutant (∆*crhABDEFG*), TLF65 *(*∆*crhDEF*) was co-transformed with respective pFC332_*crh*-sgRNA plasmids and corresponding knockout-repair DNA fragments. Simultaneous deletion of either *crhA* and *crhB* or *crhA* and *crhG* were both found to be 100% efficient in the tested transformants (*crhA* and *crhB* (7/7) and *crhA* and *crhG* (4/4), Additional file 3: Figure S3). Concurrent deletion of *crhA, crhB* and *crhG* resulted in eight transformants that were all tested for successful knockouts of all three genes. Three out of eight transformants were found to be *ΔcrhA, ΔcrhB* and *ΔcrhG* (∆*crhABDEFG*). In one transformant both *ΔcrhB* and *ΔcrhG* were deleted (∆*crhBDEFG*), whereas the four other transformants were deleted for *ΔchrA* (Additional file 3: Figure S3). The observed reduction in knockout efficiency among transformants marks a tipping point at which the attempt can be considered worthwhile. Note however that, despite a significantly lower efficiency score compared to double knockouts, the set-up with three individual gene targeting plasmids—each with identical selection markers and separate knockout-repair DNA fragment—remains within practically manageable numbers of strains to be analyzed.

**Fig. 1:**
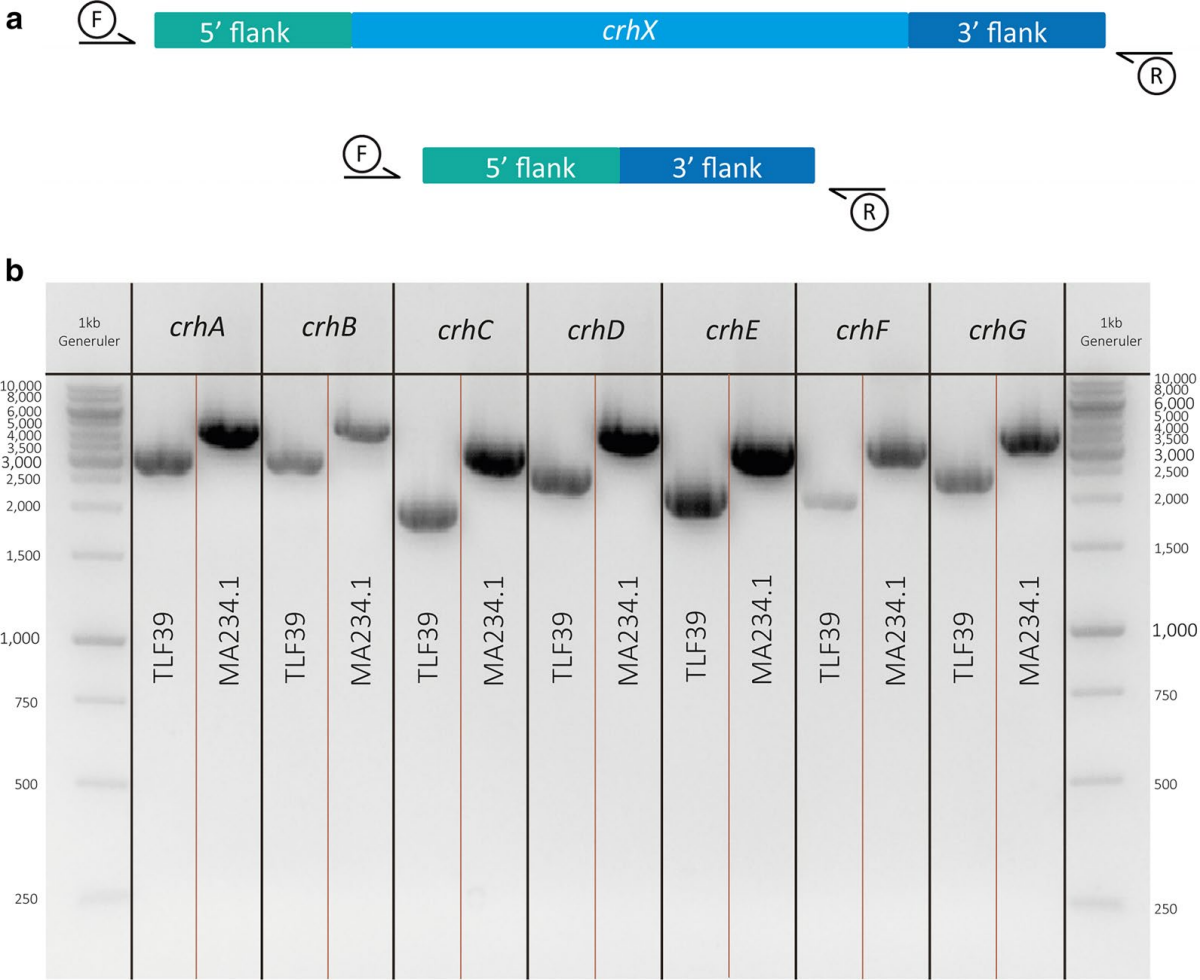
Diagnostic PCR of *crhA*-*G* in the *A. niger* TLF39 and wild type (MA234.1) strains. ORFs removed for each *crh* gene in TLF39 show a downward band shift compared to MA234.1. **a** Exact band sizes are listed. **b** gDNA of TLF39 and MA234.1 was amplified with primer pairs for each *crh* gene (listed in Additional file 4: Table S1); PCR samples were loaded on 1% agarose gels

Lastly, ∆*crhABDEFG* (TLF68) was transformed with pFC332_*crhC*-sgRNA and respective knockout-repair DNA fragment yielding a septuple ∆*crhABCDEFG* knockout (TLF39) of the whole *crh* gene family. A diagnostic PCR (primers used are listed in Additional file 4: Table S1) of the each *crh* gene shows a band size difference between wild type strain and TLF39 (Fig. 1a). Results of the diagnostic PCR of both the mutant (TLF39) and wild type (MA234.1) strains are shown in Fig. 1b, where all ORFs of the seven *crh* genes are removed resulting in a size difference.

**Fig. 2:**
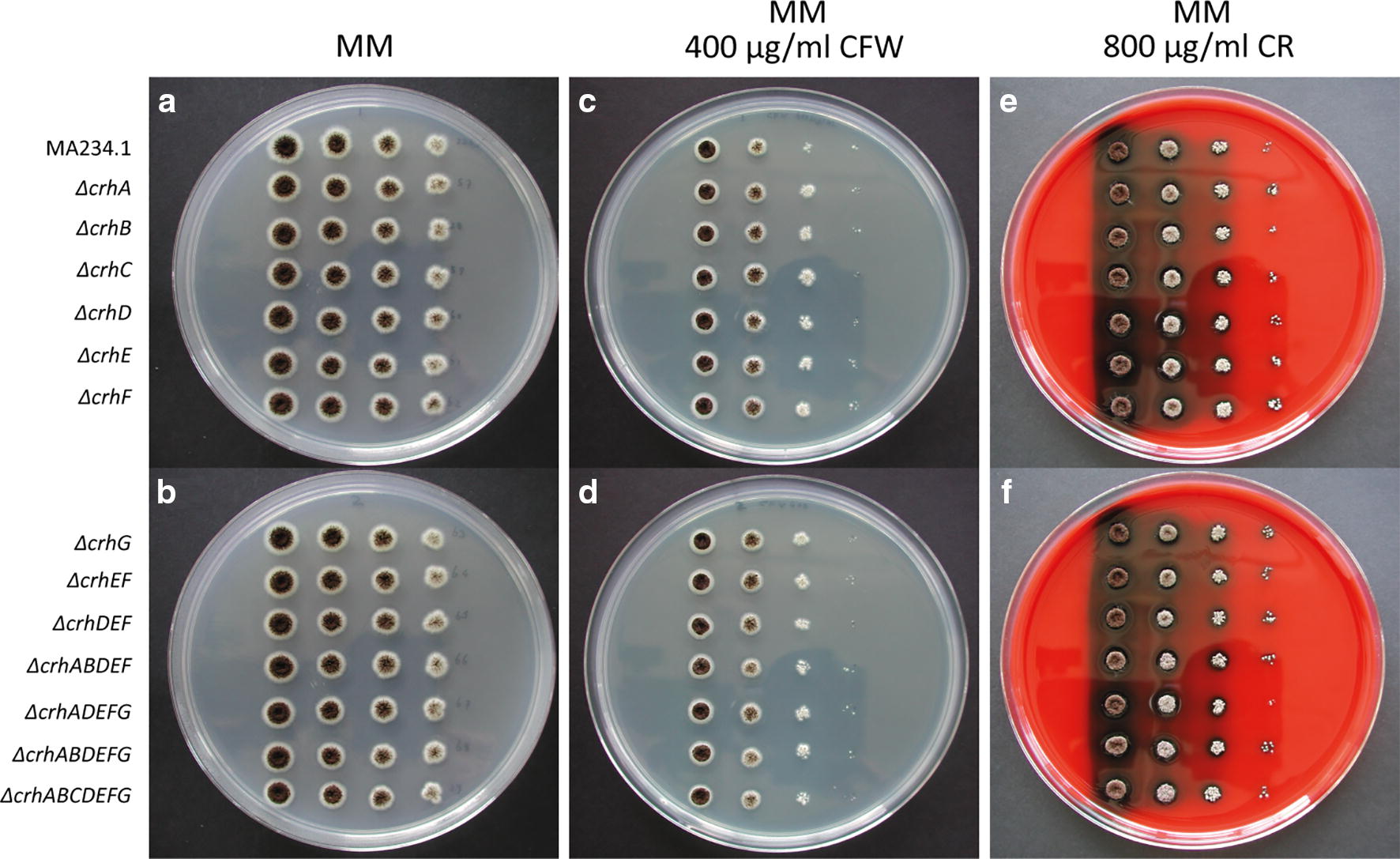
Growth morphology of *crh* knockouts in MA234.1 (*ΔkusA*) obtained using CRISPR/Cas9. MA234.1, *ΔcrhA, ΔcrhB, ΔcrhC, ΔcrhD, ΔcrhE, ΔcrhF, ΔcrhG, ΔcrhEF, ΔcrhDEF, ΔcrhABDEF, ΔcrhADEFG, ΔcrhABDEFG and ΔcrhABCDEFG* on MM (**a**, **b**), MM + 400 µg/mL CFW (**c**, **d**) or MM + 800 µg/mL CR (**e**, **f**)

All single, intermediate triple, quintuple and final septuple CRISPR/Cas9 derived *crh* knockouts were spotted on MM 1% glucose. As is evident from Fig. 2, there is no difference in growth between the different knockouts. However, there is a subtle difference in compactness of the colony morphology between the wild type and the septuple *crh*–knockout (Fig. 2a, b).

### *Crh* gene knockouts via split marker transformations and effects of the integrated *AOpyrG* marker

At the time of setting up CRISPR/Cas9 based gene editing in *A. niger,* we used traditional split marker (i.e. bipartite) transformation to assess the effects of single gene deletions of the *crh* gene family. All seven single knockouts of *crhA*-*G* were obtained using the auxotrophic *Aspergillus oryzae pyrG* (*AOpyrG*) selection marker in a ∆*kusA, pyrG*- background (MA169.4, Table 2). To allow possible future transformation of single gene knockouts we opted to use direct-repeat (DR) split marker flanks required for looping-out the marker via counter selection (see “Methods”). However, synthesis of DR split marker flanks proved to be problematic for *crhG* and forced us to refrain from creating a *ΔcrhG*::DR-*AOpyrG*-DR type construct (data not shown) and to use a *ΔcrhG*::*AOpyrG* deletion construct instead. Transformant colonies were single streaked for purification, and genotyping through diagnostic PCR showed correct replacement of the ORF by *AOpyrG* for all seven genes (data not shown). During single streaking for strain purification it became evident that *ΔcrhG*::*AOpyrG* transformants already showed poor growth on MM (Additional file 5: Figure S4), whereas CRISPR/Cas9 constructed ∆*crhG* was not found to display a growth phenotype (Fig. 2b). Supplementation of the medium with 10 mM uridine (auxotrophic *pyrG*- supplementation) abolished the poor growth phenotype for ∆*crhG*::*AOpyrG* compared to wild type strain MA234.1 (Additional file 5: Figure S4), indicating that poor growth was due to insufficient levels of endogenously synthesized uridine/uracil.

All remaining single knockouts (∆*crhA*-F) were cultured to obtain fresh spore solutions and were spotted in equal spore numbers on MM with cell wall disturbing compounds Calcofluor White (CFW) and Congo Red (CR), to assess phenotypic effects (MM + CFW and MM + CR). Wild type MA234.1 and mutants were cultured under equal conditions (Additional file 6: Figure S5). No growth effects were found for ∆*crhA*::DR-*AOpyrG*-DR, ∆*crhB*::DR-*AOpyrG*-DR, ∆*crhC*::DR-*AOpyrG*-DR, ∆*crhD*::DR-*AOpyrG*-DR and ∆*crhF*::DR-*AOpyrG*-DR on both MM, MM + CFW and MM + CR. However, ∆*crhE*::DR-*AOpyrG*-DR only displayed a growth phenotype on MM containing either CFW or CR, but not on MM alone (Additional file 6: Figure S5). Similar to the situation of ∆*crhG*::*AOpyrG*, uridine supplementation to MM + CFW and MM + CR plates abolished the growth phenotype for ∆*crhE*::DR-*AOpyrG*-DR (Additional file 7: Figure S6). Set aside, our data show that single knockouts of *crh* genes do not affect growth or morphology per se, analogous to CRISPR/Cas9 obtained single mutants.

### Effects on cell wall sensitivity in crh-knockouts derived from CRISPR/Cas9 mediated transformation

To follow up on the phenotypic analysis of *crh* knockouts in bipartite-obtained transformants, CRISPR/Cas9-derived transformants were also tested for cell wall sensitivity in accordance with yeast literature on CRH functionality [5], using MM with CFW or MM with CR. In addition to previously reported expression data on plates (Table 3), we checked expression of *crh* genes in the *ΔugmA* (UDP-galactopyranose mutase A) strain [31]. The *ΔugmA* strain is known to exhibit a constitutive state of cell wall stress and may therefore resemble conditions similar to cell wall stress induced by either CFW or CR. Expression data of the *ΔugmA* strain indicates a 2.91 log2 fold-change of *crhE* under cell wall stress conditions, whereas additional positive differential expression of both *crhA* and *crhF* and negative differential expression of *crhB* are noticeable, but not scored as significant (data not shown). Consequently, we postulate that *crh* genes are required in the cell wall stress response and that deletion mutants phenotypes reflect this in a cell wall stress assay with CFW or CR. Phenotypes on MM with either CFW or MM with CR of CRISPR/Cas9 derived single knockouts and the septuple knockout of *crhA*-*G* are shown in Fig. 2c–f. It is clear from the assay that both single knockouts and the septuple knockout of *crhA*-*G* do not show a disturbed growth phenotype on MM, and show no sensitivity towards either of the tested compounds. These data suggest that the knockouts—both in singular cases and in septuple combination—do not affect the ability of *A. niger* to grow. Moreover, CrhA-G do not appear to be required for the survival when the cell wall is stressed with either CFW or CR.

## Discussion

This study demonstrates an efficient CRISPR/Cas9 based gene-editing procedure for *A. niger* using a combination of the previously described methods [20, 32]. Our approach combines the use of the pFC332 Cas9-AMA1 vector with the Pro^1^-sgRNA expression cassette. Due to a unique *Pac*I site located on the pFC332 plasmid, newly synthesized sgRNA expression cassettes could easily be ligated to generate pFC332_sgRNA plasmids both quickly and effectively. These plasmids have been shown to generate either single, double or triple knockouts in combination with homologous knockout-repair DNA fragments in high frequencies, allowing fast and efficient construction of multiple gene knockouts in a marker-free way. Such gene alterations can be performed in any strain devoid of a hygromycin selection marker, and circumvents the reliance on auxotrophic mutant strains (e.g. *AOpyrG*-). Moreover, the curability of these hygromycin containing AMA1-vectors in the absence of selection pressure as an intrinsic property warrants the recyclability of this system for putative recurrent transformations, without the need to use of possibly mutagenic counter selection strategies.

An often overlooked limitation of CRISPR/Cas9 based gene editing is that Cas9–sgRNA functionality is difficult to predict. In our approach, the use of the *ΔkusA* strain allows for sufficient assessment of this functionality; in the absence of a repair DNA fragment, an efficient sgRNA does not result in any transformants on the transformation plates due to the inability to repair a Cas9 induced DSB. This was observed for six out of the eight sgRNAs presented here. In two instances, transformants were observed when no knockout-repair DNA fragment was supplied, which may suggest less efficient generation of DSBs. However, in these two occasions the knockout efficiency was still 80-83% through integration of the knockout-repair DNA fragment (Table 4). These data show that sgRNAs are effectively expressed using our procedure.

As an alternative strategy to homology directed DNA repair editing, gene disruptions can be made in a *kusA*+ wild type strain without the addition of repair DNA fragment. Selection for indels is enforced by recurrent recognition and cutting by the Cas9-gRNA complex of the targeted DNA. As such, this would provide a more simplistic model for gene disruption. Song et al. [20] have previously shown the occurrence of differently sized indels in either *fwnA* (NRRL3_00462, An09g05730) or *olvA* (NRRL3_01039, An14g05350) in *A. niger* NRRL3, ranging from 88 bp insertions up to 1096 bp deletions across 30 individual mutants. In this study, while targeting the *brnA* gene, we observed even larger deletions that potentially range over 2 kb (data not shown). The DSB in *brnA* in the wild type strain N402 caused by pFC332_*brnA*-sgRNA lies 2.0 kb upstream from the *olvA* start codon. *OlvA* is epistatic over *brnA* in the melanin synthesis pathway [26, 27], and a knockout of *olvA* becomes olive-colored. N402 transformed with pFC332_*brnA*-sgRNA occasionally (2.8%, n = 580) showed olive-colored transformants (data not shown), suggesting that deletions extend over previously reported 1 kb. As a result, we avoid this type of gene disruption due to the unpredictable outcome, and instead, highly recommend to perform gene edits with targeted repair DNA fragments in *ΔkusA* background.

In addition to creating single knockouts with Cas9–sgRNA plasmids, we have shown to efficiently multiplex two or three knockouts using identical vectors (pFC332-Cas9 backbone), harboring separate gRNAs, in a single transformation. These results suggest that multiple, different AMA1-vector copies with the same selection marker can successfully create knockout combinations in *A. niger*. This positive result contradicts findings in *A. oryzae* where multiplexing with two different sgRNA on separate AMA1-vectors only resulted in single knockouts [33]. Only when both sgRNA constructs were cloned into a single vector, similar double knockout efficiencies observed as described here. It remains to be tested to what extent this number of simultaneous gene edits can be achieved in *A. niger*, but may ultimately be limited to the maximum copy number of approximately 10–20 AMA1 plasmids per nucleus [34].

The CRISPR/Cas9 procedure presented here provides evidence that multiplexing up to three targets does not require expression of three different sgRNAs on a single plasmid. High knockout efficiencies can be acquired by combining individual gene targeting plasmids with respective knockout-repair DNA fragments. This allows to combine different gene targeting plasmids as desired. Hence, existing plasmids can be used in various combinations without the need for reconstruction of complex cassettes, such as described by Nødvig et al. [21]. In addition, we show that multiple genetic alterations, such described for multiple gene knock-ins [17], do not need integrative selection markers to ensure efficient genome editing. This work has addressed the current limitations of creating marker-free multiplex knockouts of separate genes in *A. niger*, without the reliance on either integrative plasmids [14, 19] or integrative (albeit recyclable) selection markers [15–18], and can directly be re-transformed for additional gene editing if desired. Therefore, it can be concluded that the use of singular sgRNA expression cassettes provides a flexible system in which both single, double and triple marker-free knockouts can efficiently be made.

CRISPR/Cas9 based genome editing is now a relevant technique in addition to conventional split marker transformations. The benefit of using CRISPR/Cas9 is multiplex ability, independence of integrative selection markers and the option to reuse the identical selection marker in follow-up gene-editing steps. Currently, recyclable systems do exist for *pyrG* and *amdS* in both CRISPR/Cas9 gene editing [17, 18] and conventional split marker transformation [11]. This process can be induced with the counter-selectable compounds, either 5-FOA or 5-FAA, for *pyrG* and *amdS* markers respectively. Exposure to 5-FOA/FAA selects for either loop-out in case of direct repeat elements or loss (of function) of *pyrG/amdS*, as the active gene metabolizes 5-FOA/FAA to a toxic compound. Consequently, the *pyrG/amdS* gene can be recycled to target a different GOI. In this way, any gene can be systematically knocked out in a relatively time consuming single-step fashion. In addition to being more labor intensive, successive use of 5-FOA/FAA may be undesirable due to their mutagenic nature. To put this in perspective, we estimate that construction of a septuple knockout strain can be obtained 3 times faster with CRISPR/Cas9 gene editing than split marker *pyrG* recycling due to the high efficiency of multiplexing and the omission of the counter selection procedure.

In addition to CRISPR/Cas9 obtained *crh* knockouts, single knockouts were constructed using the recyclable *pyrG* marker to test Crh functionality. We observed an effect from the locus of integration for the *AOpyrG* selection marker in case of *crhG,* which is most likely related to low baseline expression (Table 3). Deletion of *crhG* via *AOpyrG* integration resulted in a poor growth phenotype on MM, and was shown to be complemented with the addition of uridine (Additional file 5: Figure S4). Additionally, unlike *crhG*, ∆*crhE*::*DR*-*AOpyrG*-*DR* shows normal growth on MM, but shows impaired growth on MM with either cell wall disturbing compounds CFW or CR. Cell wall stressing compounds CFW and CR are known disrupt fungal cell wall assembly [35], triggering the cell wall integrity (CWI) response [36]. In addition to chemical disruption, the CWI pathway is constitutively activated in the *ΔugmA* mutant, lacking cell wall galactofuranose [37]. This condition resembles exposure to CFW or CR, and transcriptomic analysis of the *ΔugmA* mutant showed upregulation of *crhE,* suggesting its importance during the CWI stress response. However, parallel phenotypic analysis of MA632.2 (∆*crhE*::*DR*-*AOpyrG*-*DR*) with the CRISPR/Cas9 derived TLF61 (∆*crhE*) showed no affected growth phenotype for TLF61 when exposed to either CFW or CR, whereas MA632.2 showed sensitivity toward both compounds. Only when the medium was supplemented with uridine, we observed the same wild type like phenotype as TLF61 (Additional file 7: Figure S6). Despite the projected hypothesis on the requirement of *crhE* in the CWI response, these findings indicate an effect on the *AOpyrG* marker during the CWI response rather than the lack of CrhE. A possible explanation may be the locus activity of *crhE* (e.g. chromatin remodeling) during cell wall stress, affecting expression of ectopically integrated genes such as *AOpyrG* in MA632.2.

Ectopic expression of auxotrophic markers have previously been described to be affected by the locus of integration. Specifically, the use of *pyrG* marker was reported to have a negative effect on either the ability to have a proper sexual cycle in *A. nidulans* [25] or during vegetative growth in *Aspergillus flavus* [38]. In case of *A. nidulans*, Robellet and colleagues clearly showed lower expression of *pyrG* at the *alcS* locus (required for ethanol utilization) compared to other ectopic integration sites of *pyrG.* This was tested in both *alcS*-locus inducing and non-inducing conditions, and may thus not only be related to an active or silent locus, but also where the position of the locus resides on the genome. These “position effects”, where the expression of the selectable marker highly depends on the genetic elements at the locus of integration [39], have been also been reported for *pyr4* (*pyrG* orthologue) in *N. crassa*, *nadA* and *argB* in *A. nidulans* [22–24] and URA3 (*pyrG* orthologue) in *Candida albicans* [40, 41]. Taken together, these reports highlight that the expression of integrative selection markers, and specifically *pyrG*/*pyr4*/*URA3*, are sensitive to the locus of integration. In this study, we found that the auxotrophic *AOpyrG* selection marker is affected by the locus of integration. Therefore, caution must be taken in interpreting the phenotypic and/or pleiotropic effects that arise from this artifact in strains that harbor integrative selection markers. We propose to use either alternative, dominant selection markers such as hygromycin for construction of single knockouts or use non-integrative CRISPR/Cas9 selection procedures such as the one presented here.

## Conclusions

We have demonstrated the efficiency of marker-free multiplex gene knockout construction, using Cas9–sgRNA plasmids with marker-free repair DNA fragments, in parallel to split marker fragment transformation to knockout the cell wall chitin cross-linking gene family (*crhA*-*G*). The use of multiple Cas9–sgRNA plasmids harboring the same selection marker can be achieved 3 times faster than *AOpyrG* recycling; showing that double or even triple knockouts are possible at relatively high efficiency using CRISPR/Cas9. Moreover, removal of selection medium allows the loss of Cas9–sgRNA plasmids while the gene knockout remains present. In turn, this grants the recurrent use of plasmids with the same selection marker in future transformations without prior need to recycle *pyrG/amdS*-type integrative selection markers. Additionally, it became evident that the expression of the integrated *AOpyrG* selection marker was affected in two of the seven *crh* knockout strains generated by a classical split marker approach compared to the same gene knockouts in CRISPR/Cas9 obtained mutants. Therefore, the marker-free CRISPR/Cas9 procedure presented here clearly favors over integrative selection marker-based transformations formultiplex knockout strain construction.

## Methods

### Strains, media, growth conditions and transformations

*Aspergillus niger* strains MA234.1 (*cspA1, ΔkusA::DR*-*amdS*-*DR*) [31] and MA169.4 (*cspA1, ΔkusA::DR*-*amdS*-*DR, pyrG*) [42] were used in this study. Strains used in this study can be found in Table 2. All media were prepared as described by Arentshorst et al. [11]. In all cases minimal medium (MM) contained 1% (w/v) glucose, 1.5% agar and was supplemented when required with either uridine (10 mM) or hygromycin (100 µg/mL). Complete medium (CM) contained 1% (w/v) glucose, 1.5% agar (Scharlau, Barcelona, Spain), 0.1% (w/v) casamino acids and 0.5% (w/v) yeast extract in addition to MM. To harvest spores, strains were first inoculated from − 80 °C glycerol stocks onto fresh CM plates and were allowed to grow and sporulate for 5–7 days at 30 °C. Spores were harvested by addition of 15 mL of 0.9% (w/v) NaCl to CM spore plates and were gently scraped from the plate surface with a cotton stick. Spore solution was pipetted through sterile cotton filters (Amplitude™ Ecocloth™ Wipes, Contec Inc., Spartanburg, SC, USA) to eliminate large mycelial debris.

Strains were transformed after protoplastation as described previously [11]. We used 2 µg of Cas9–sgRNA plasmid with approximately 2 µg of repair DNA fragment (1.8–2.0 kbp) for each transformation. Transformation plates were incubated on MMS containing hygromycin (200 µg/mL) for 6 days at 30 °C. Transformed colonies were single streaked on MM containing hygromycin (100 µg/mL) to ensure nuclei of spores harbor the Cas9–sgRNA plasmid, thus are most likely to be transformed. Next, a single colony was picked and transferred to non-selective MM medium to allow loss of the Cas9–sgRNA plasmid. A third streak of a single colony on both MM containing hygromycin (100 µg/mL) and MM acts as a control for loss of plasmid. DNA from plasmid-cured strains was isolated as described by Arentshorst et al. [11], using mortar and pestle to grind the mycelium in liquid nitrogen.

### CRISPR/Cas9 plasmid design

**Fig. 3:**
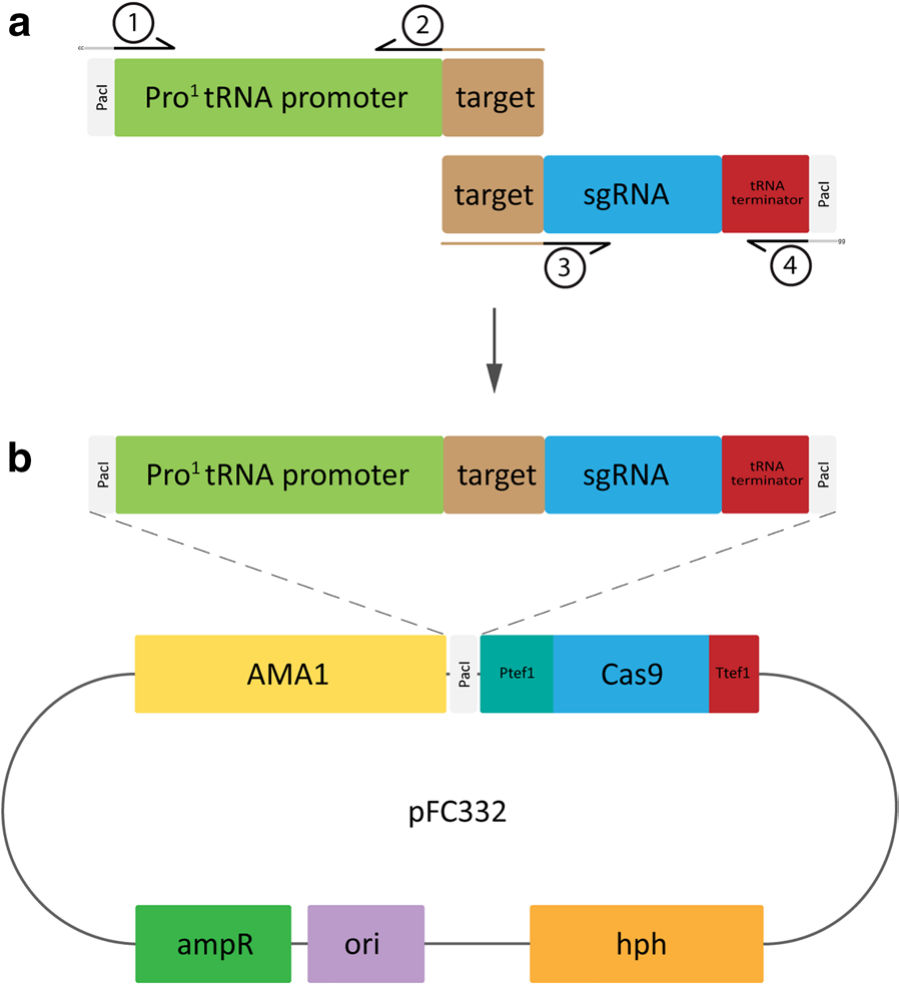
Schematic representation of the pFC332_Pro^1^-sgRNA plasmid construction. **a** Amplification of the two flanks that represent the Pro^1^-sgRNA expression cassette: pTE1_for and pRC-target are used to amplify the Pro^1^-tRNA promoter and target sequence, where pRC-target contains a variable 20 bp overhang (indicated by brown color) that represents the reverse complement target sequence of choice. In turn, pTarget and pTE1_rev are used to amplify the target-sgRNA-Pro^1^-tRNA terminator flank. Here, pTarget contains a variable overhang that contains the target sequence of choice. Separate flanks are joined together through fusion PCR by pTE1_for and pTE1_rev, where the overhang sequence (=target) facilitates the homologous region between both flanks. **b** Addition of *Pac*I sites to either end of the fusion construct (part of pTE1_for and pTE1_rev sequence) allows ligation of the fusion construct into pFC332. Diagnostic restriction analysis of the cloned plasmid ought to be done with *Cfr*42i (*Sac*II) and shows a fragment of either 497 bp or 500 bp in addition to 1 kb and 14.3 kb fragments, for forward or reverse orientation, respectively

To express Cas9 and the guide RNA from the same autonomously replicating vector using hygromycin as a selection marker for fungal transformation, plasmid pFC332 was used [32]. pFC332 contains a unique *Pac*I site which was used to insert a single guide RNA (sgRNA) expression cassette based on the native *A. niger* RNA polymerase type III Pro^1^-promoter and terminator [20]. The PCR strategy to generate sgRNA expression cassettes is schematically shown in Fig. 3. The strategy is based on designing overlapping PCR fragments containing the Pro^1^-promoter region followed by the reverse complemented sgRNA target, and a second PCR fragment containing the target followed by the sgRNA and terminator regions. Complementary target sequences of both PCR products allow fusion through PCR with outer primers (pTE1_for and pTE1_rev) to amplify the entire sgRNA expression cassette. These primers introduce a *Pac*I site on either end of the construct for cloning into pFC332 (Fig. 3b).

Template plasmids for amplification of new sgRNA expression cassettes were made by amplifying the Pro^1^-promoter from ANEp8_Cas9_sgRNA-albA [20] with pTE1_for (Fig. 3a) to include *Pac*I restriction site with a CC overhang (5′–CCttaattaa–3′) and OTL487. Resulting PCR product (*Pac*I::Pro^1^-promoter) was cloned directly into pJET1.2/blunt cloning vector (Thermo Scientific™) to yield pTLL108.1. Similarly, the sgRNA::Terminator was amplified through PCR from ANEp8_Cas9_sgRNA-albA with OTL488 and pTE1_rev (Fig. 3) to include *Pac*I restriction site and CC overhang (sgRNA::Terminator::*Pac*I), and was cloned into pJET1.2/blunt to yield pTLL109.2 (data not shown).

The sgRNA targets were designed using the CHOPCHOP web-tool [43]. Putative targets were obtained using the *A. niger* NRRL3 genomic sequence of the gene of interest (GOI) submitted in Fasta format with default CRISPR/Cas9 settings. The *A. niger* was used as reference genome for off-target matching. A selected, variable target sequence of 20 bp (without 5′ NGG 3′ PAM sequence) was added as overhang to target specific primer pTarget (Fig. 3a) in the identical forward orientation, whereas the reverse complement (RC) target sequence was added as overhang to target specific primer pRC-target (Fig. 3a).

The sgRNA expression cassette was acquired through fusion of two PCR products, the 5′ flank and the 3′ flank. pTE1_for (5′–ccttaattaaACTCCGCCGAACGTACTG–3′) was used in combination with pRC-target on template plasmid pTLL108.1 to produce a 264 bp *Pac*I::Pro^1^-promoter::target sequence (5′ flank). Combined primer pair pTarget and pTE1_rev (5′–ccttaattaaAAAAGCAAAAAAGGAAGGTACAAAAAAGC–3′) was used on template plasmid pTLL109.2 to create the 133 bp target::trRNA::Terminator::*Pac*I sequence (3′ flank). After gel purification, the 5′ flank was subsequently fused to the 3′ flank, facilitated by the unique target as complementary sequence between the two flanks. Amplification of both flanks in one reaction with pTE1_for and pTE1_rev (Fig. 3b) results in a 397 bp sgRNA construct *Pac*I::Pro^1^-promoter::target::trRNA::Terminator::*Pac*I (Fig. 3b). PCR conditions: 5× HF buffer (Phusion), 200 µM dNTP mix (final conc.), 0.2 µM of each primer (final conc.), 2 ng template plasmid (pTLL108.1 or pTLL109.2), 0.1 µL/10 µL reaction volume of Phusion Polymerase (Thermo Scientific™). PCR cycle settings: 30 s. initial denaturation (98 °C), 5 s. denaturation (98 °C), 5 s. annealing (60 °C), 6 s. extension for 5′ flank, 2 s. extension for 3′ flank and 12 s. for fusion PCR of both flanks (72 °C), repeat denaturation to extension cycle 30×, final extension 30 s. for individual flanks, 2 min. for fusion PCR (72 °C), hold at 10 °C.

### Plasmid construction and repair DNA fragment design

sgRNA constructs that were obtained through PCR were column purified (GeneJET PCR Purification Kit, Thermo Scientific™) and digested with *Pac*I overnight at 37 °C for 16 h and inactivated for 20 min. at 80 °C in a thermocycler (set heated lid to 37 °C). Digested samples were column purified and stored on ice prior to ligation. Approximately 1 µg of pFC332 was digested with *Pac*I at 37 °C for 1 h and heat inactivated at 80 °C for 20 min. in a water bath, prior loading the entire sample on gel. Excision of linearized pFC332 plasmid (15.6 kb) followed by gel purification (GeneJET Gel purification Kit, Thermo Scientific™) was eluted in 30 µL MQ. Linearized plasmid (17 µL) was dephosphorylated in a total volume of 20 µL with FastAP (FastAP Thermosensitive Alkaline Phosphatase (1 U/µL), Thermo Scientific™) for 10 min. at 37 °C, followed by 10 min. inactivation at 75 °C. Samples were put directly on ice after inactivation. Treated samples were not purified prior to ligation: 3 µL plasmid together with 12 µL sgRNA construct was used in a ligation reaction for 10 min. (Rapid Ligation Kit, Thermo Scientific™). Entire ligation mix was transformed to competent *E. coli* DH5α cells via heat shock protocol, and cells were plated on LB containing 100 µg/mL ampicillin. Plasmids were isolated from successful transformants according to Miniprep protocol (GeneJET Plasmid Miniprep Kit, Thermo Scientific™). Diagnostic digest with *Cfr*42i (*Sac*II) results in three bands of approximately, 14.3 kbp, 1.1 kbp and 500 bp in case of sgRNA integration, whereas in case of a control digest of pFC332 (or empty transformed vector), the 500 bp band will be absent. Confirmed plasmids were sent for Sanger sequencing with pTE1_for (Macrogen Europe, Amsterdam, The Netherlands).

**Fig. 4:**
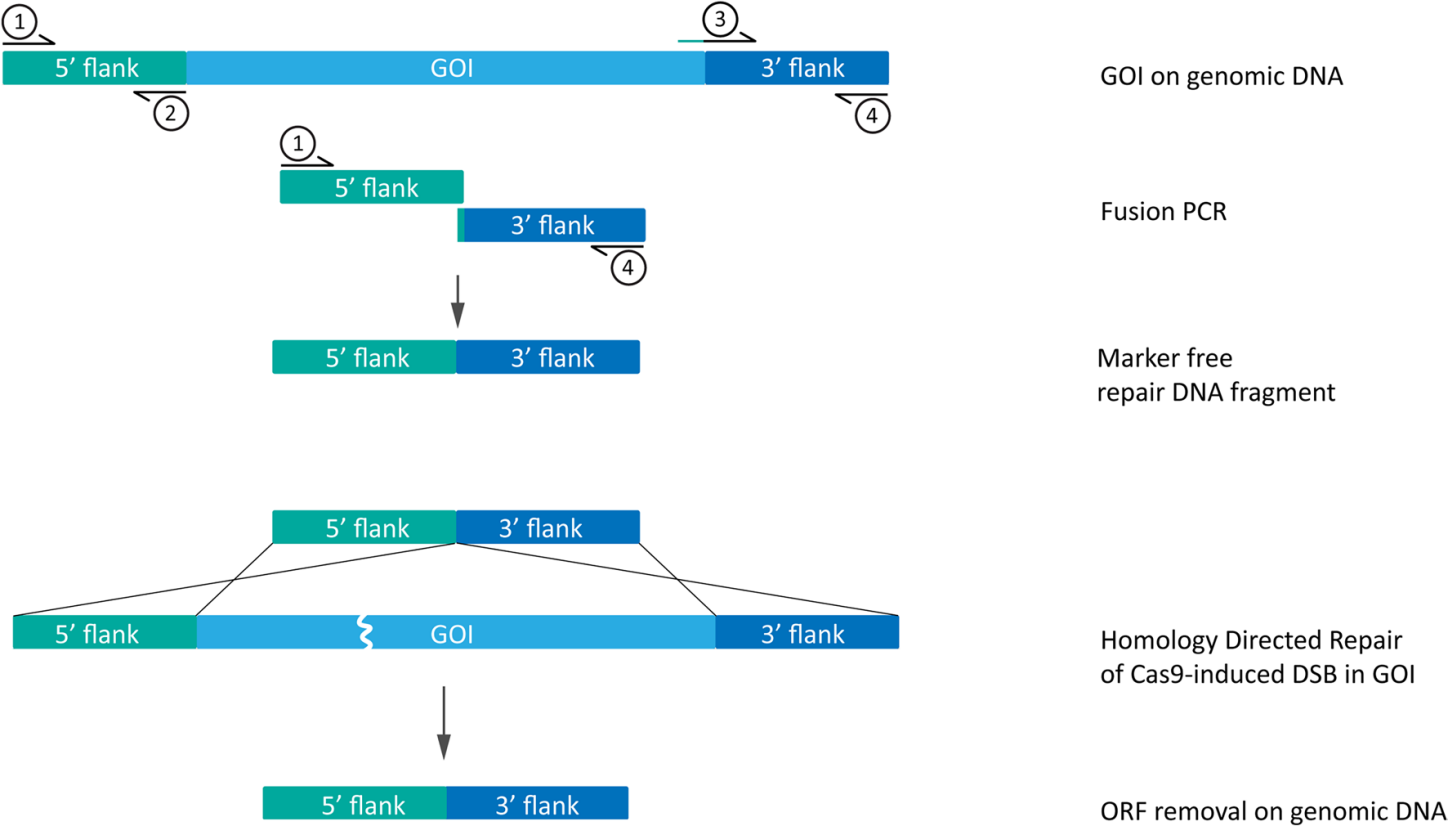
Construction of marker free repair DNA fragment. Amplification of regions upstream (5′ flank) and downstream (3′ flank) of a gene of interest (GOI): Primer 1 and Primer 3 are used to amplify the 5′ flank, typically directly upstream from the start codon of the GOI. Primer 2 and Primer 4 are used to amplify the 3′ flank, just downstream of the ORF stop codon. Addition of 20 bp reverse complement sequence of Primer 3 to the 5′ end of Primer 2 ensures overlap between the 5′ and 3′ flanks necessary for fusion of the two flanks to construct the marker free repair DNA fragment. Upon introduction of the marker free repair DNA fragment to the fungal cell, repair of the double strand break (DSB) induced by a Cas9–sgRNA complex is possible by homology directed repair (HDR) at the site of the GOI

Repair DNA fragments to create gene knockouts were based on a similar concept as producing split marker flanks in bipartite transformation [11]. Flanking regions on both 5′ and 3′ end around the gene of interest (GOI) were selected to be approximately 800–1000 bp each. The forward primer on the 3′ repair flank (Primer 3, Fig. 4) was provided with an overhang sequence which is the reverse complement sequence of the reverse primer on the 5′ end (Primer 2, Fig. 4). This created a 20 bp overlap between 5′ and 3′ repair flanks, required for fusion PCR (Fig. 4).

### Cell wall sensitivity assays

Cell wall disturbing compounds Calcofluor White (CFW) and Congo Red (CR) were added in respective concentrations of 400 µg/mL and 800 µg/mL to MM plates [44]. Spores were counted, serially diluted into 2000, 200, 20 and 2 spores/µL and 5 µL of respective dilutions were spotted on MM plates containing either CFW or CR. Plates were incubated for 3–5 days at 30 °C.

## Supplementary information

**Additional file 1: Figure S1.** PEG-mediated transformation of the A*. niger ΔkusA* strain targeting the *brnA* gene. Protoplasts were transformed with a pFC332-Cas9 vector, either with a sgRNA expression cassette (pFC332-*brnA*_sgRNA; A and C) or without any sgRNA expression cassette (pFC332; B and D), and were grown on MM containing 32.5% (w/v) sucrose (MMS) and 200 µg/mL hygromycin B. Knockout-repair DNA fragment was either left out (A and B) or added (C and D) to the protoplasts in addition to the plasmid. (E) Single streak of a *ΔbrnA* transformant taken from the plate shown in C and (F) a transformant taken from the plate shown in D.

**Additional file 2: Figure S2.** Diagnostic PCR of the *brnA* locus. (A) Shows the forward (F) and reverse (R) primers used to amplify the *brnA* on the gDNA of *A. niger*. The PCR amplified *brnA* locus in the wild type strain is 4012 bp, whereas that in the *ΔbrnA* is expected to be 2411 bp. (B) *A. niger* gDNA was isolated and amplified with F and R primers from both MA234.1 (1), *ΔbrnA* transformants (2-10, Additional file 1: Figure S1C) and black transformants (pFC332 + knockout-repair DNA) (11-14, Additional file 1: Figure S1D). PCR samples were loaded on 1% agarose gels. Ladder: 1 kb Generuler.

**Additional file 3: Figure S3.** Diagnostic PCR of all knockout mutants created in the *A. niger* MA234.1 (*ΔkusA)* background. Single (TLF57-63), double (TLF65-67) and triple (TLF68) gene. The table on the top shows the expected PCR product sizes based on the ORFs removed with the knockout repair DNA fragments for each *crh* gene. gDNA of all mutants, wild type and a negative water control (MQ) was amplified with primer pairs for each *crh* gene listed in Additional file 4: Table S1 (“HDR check” primers). Different mutants from the same transformation plate are indicated by a number, ranging from 1-8. PCR samples were loaded on 1% agarose gels with 1 kb Generuler ladder. All correctly removed ORFs show a downward band shift compared to MA234.1 (WT). Red arrows indicate the selected mutants which are included in this study.

**Additional file 4: Table S1.** All primers used in this study.

**Additional file 5: Figure S4.** Growth morphology of *ΔcrhG::AOpyrG* knockout strain on MM and MM containing 10 mM uridine.

**Additional file 6: Figure S5.** Growth morphology of single *crh* knockout strains obtained via replacing the respective *crh* gene with DR-split marker *AOpyrG* in MA169.4; ∆*crhA*-*F* on MM, MM + 400 µg/mL CFW or MM + 800 µg/mL CR.

**Additional file 7: Figure S6.** Growth morphology of MA234.1, *ΔcrhE::DR*-*AOpyrG*-*DR* and *ΔcrhE*. (A) Strains were grown on MM, MM + 400 µg/mL CFW or MM + 800 µg/mL CR. (B) Additionally, strains were grown on MM + 10 mM uridine, MM + 10 mM uridine + 400 µg/mL CFW)and MM + 10 mM uridine + 800 µg/mL CR.

## Data Availability

All dataset(s) supporting the results of this article are included within this article and its additional files. Plasmids and strains are available upon request.
