# Supplementary figures and images for: Efficient marker free CRISPR/Cas9 genome editing for functional analysis of gene families in filamentous fungi
Source: Fungal Biol Biotechnol. 2019 Sep 21;6:13. doi: 10.1186/s40694-019-0076-7 (PMC6754632; doi:10.1186/s40694-019-0076-7)

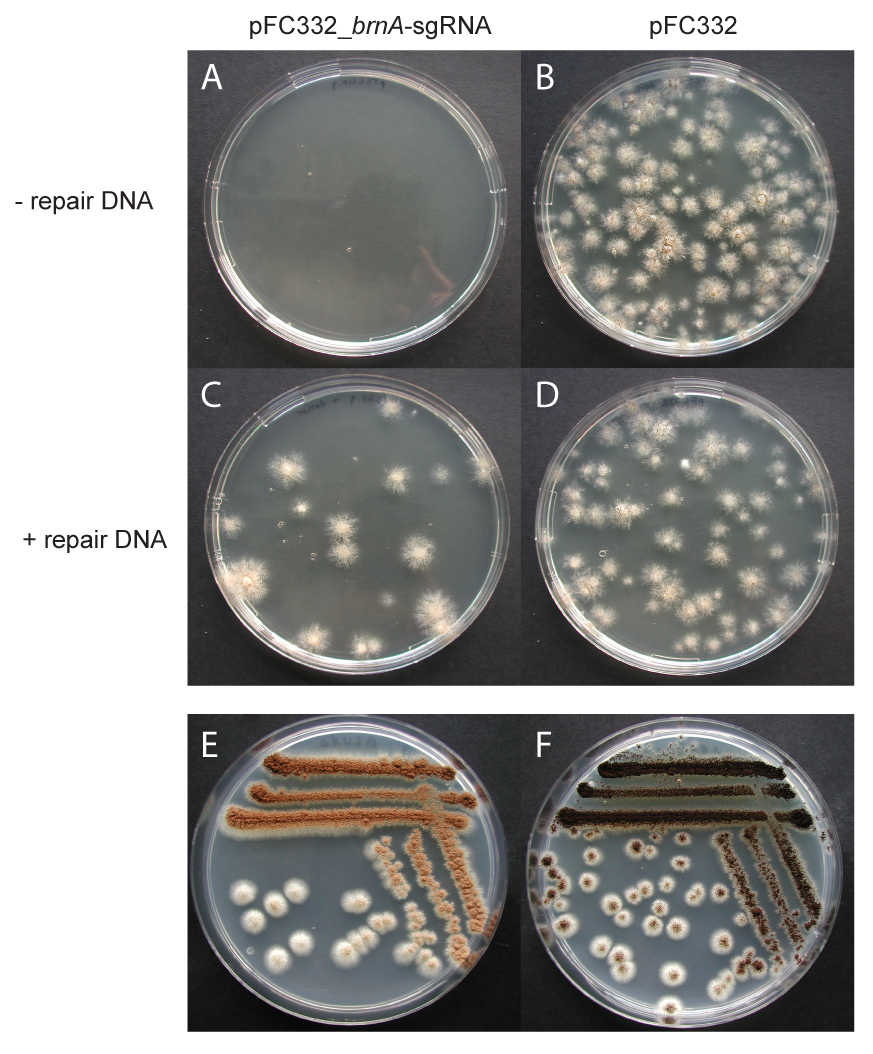

Supplement: Supplementary file 1 — Additional file 1: Figure S1. PEG-mediated transformation of the A. niger ΔkusA strain targeting the brnA gene. Protoplasts were transformed with a pFC332-Cas9 vector, either with a sgRNA expression cassette (pFC332-brnA_sgRNA; A and C) or without any sgRNA expression cassette (pFC332; B and D), and were grown on MM containing 32.5% (w/v) sucrose (MMS) and 200 µg/mL hygromycin B. Knockout-repair DNA fragment was either left out (A and B) or added (C and D) to the protoplasts in addition to the plasmid. (E) Single streak of a ΔbrnA transformant taken from the plate shown in C and (F) a transformant taken from the plate shown in D. [file 40694_2019_76_MOESM1_ESM.tif]

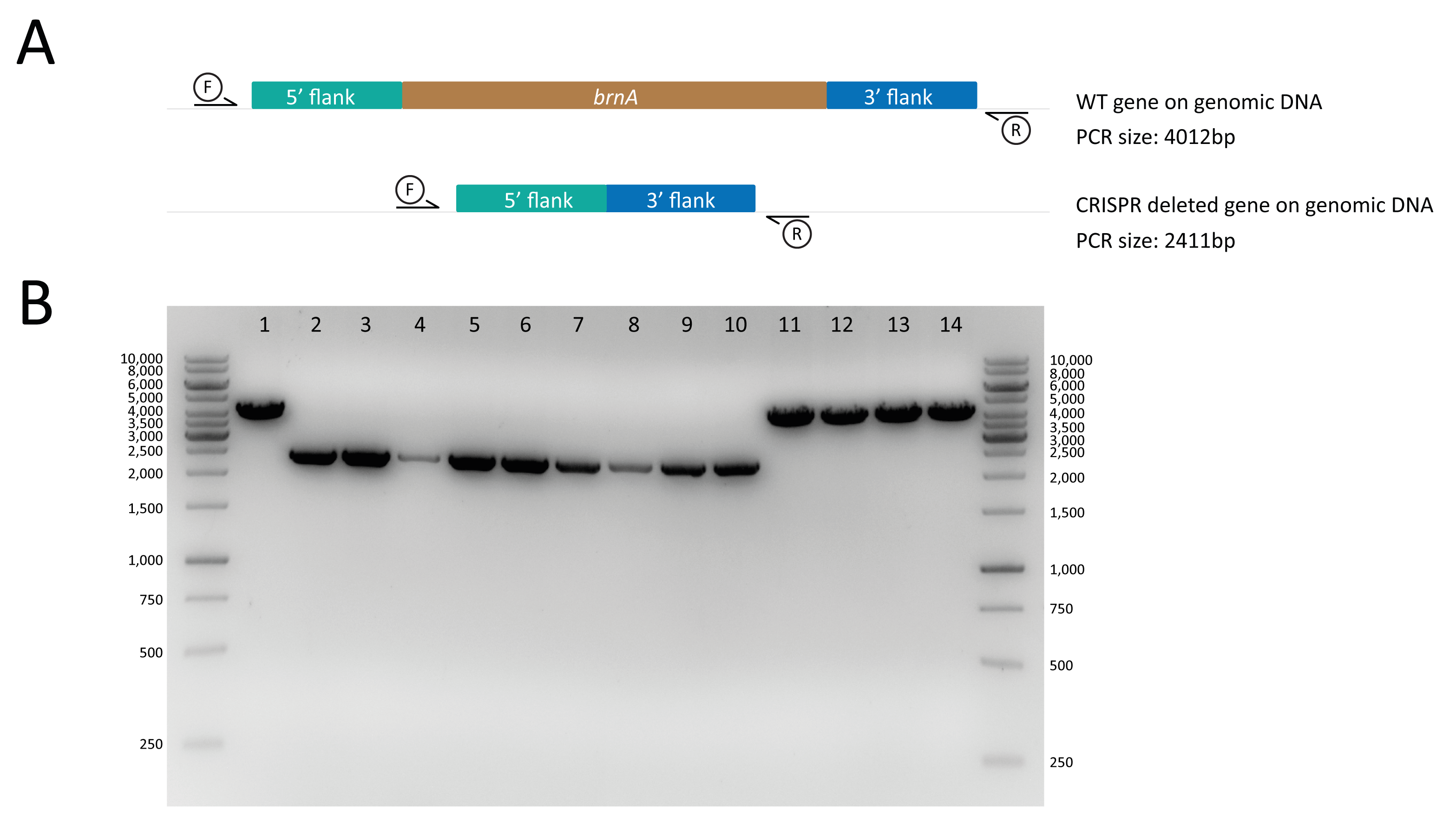

Supplement: Supplementary file 2 — Additional file 2: Figure S2. Diagnostic PCR of the brnA locus. (A) Shows the forward (F) and reverse (R) primers used to amplify the brnA on the gDNA of A. niger. The PCR amplified brnA locus in the wild type strain is 4012 bp, whereas that in the ΔbrnA is expected to be 2411 bp. (B) A. niger gDNA was isolated and amplified with F and R primers from both MA234.1 (1), ΔbrnA transformants (2-10, Additional file 1: Figure S1C) and black transformants (pFC332 + knockout-repair DNA) (11-14, Additional file 1: Figure S1D). PCR samples were loaded on 1% agarose gels. Ladder: 1 kb Generuler. [file 40694_2019_76_MOESM2_ESM.tif]

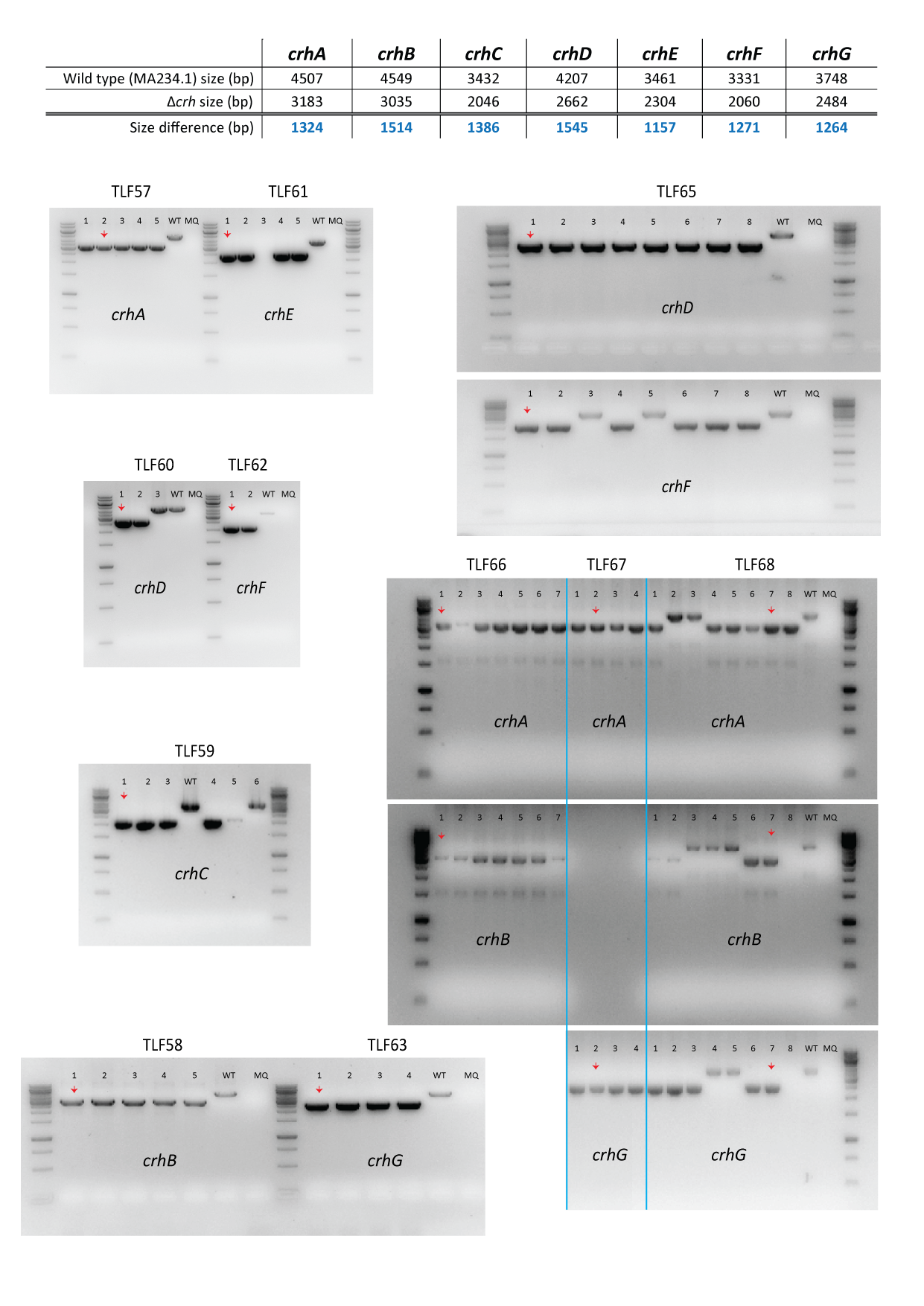

Supplement: Supplementary file 3 — Additional file 3: Figure S3. Diagnostic PCR of all knockout mutants created in the A. niger MA234.1 (ΔkusA) background. Single (TLF57-63), double (TLF65-67) and triple (TLF68) gene. The table on the top shows the expected PCR product sizes based on the ORFs removed with the knockout repair DNA fragments for each crh gene. gDNA of all mutants, wild type and a negative water control (MQ) was amplified with primer pairs for each crh gene listed in Additional file 4: Table S1 (“HDR check” primers). Different mutants from the same transformation plate are indicated by a number, ranging from 1-8. PCR samples were loaded on 1% agarose gels with 1 kb Generuler ladder. All correctly removed ORFs show a downward band shift compared to MA234.1 (WT). Red arrows indicate the selected mutants which are included in this study. [file 40694_2019_76_MOESM3_ESM.tif]

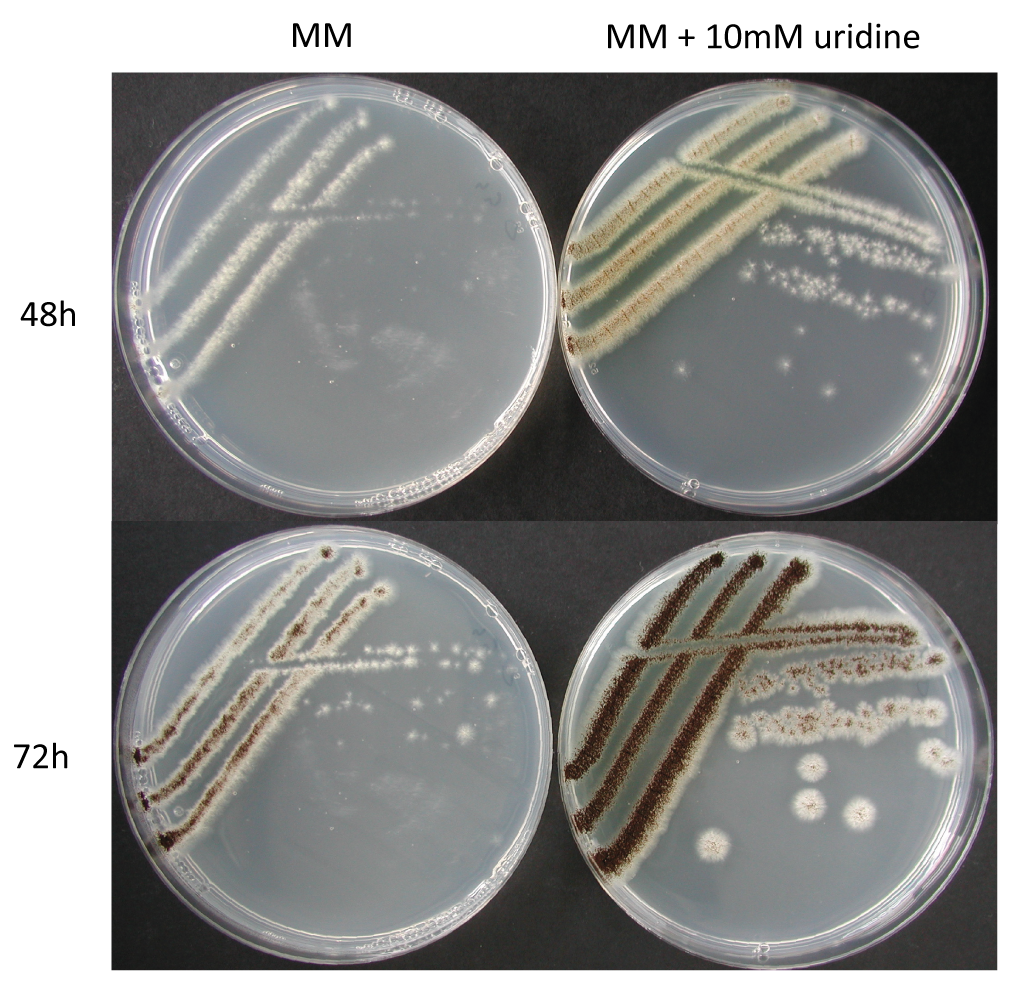

Supplement: Supplementary file 5 — Additional file 5: Figure S4. Growth morphology of ΔcrhG::AOpyrG knockout strain on MM and MM containing 10 mM uridine. [file 40694_2019_76_MOESM5_ESM.tif]

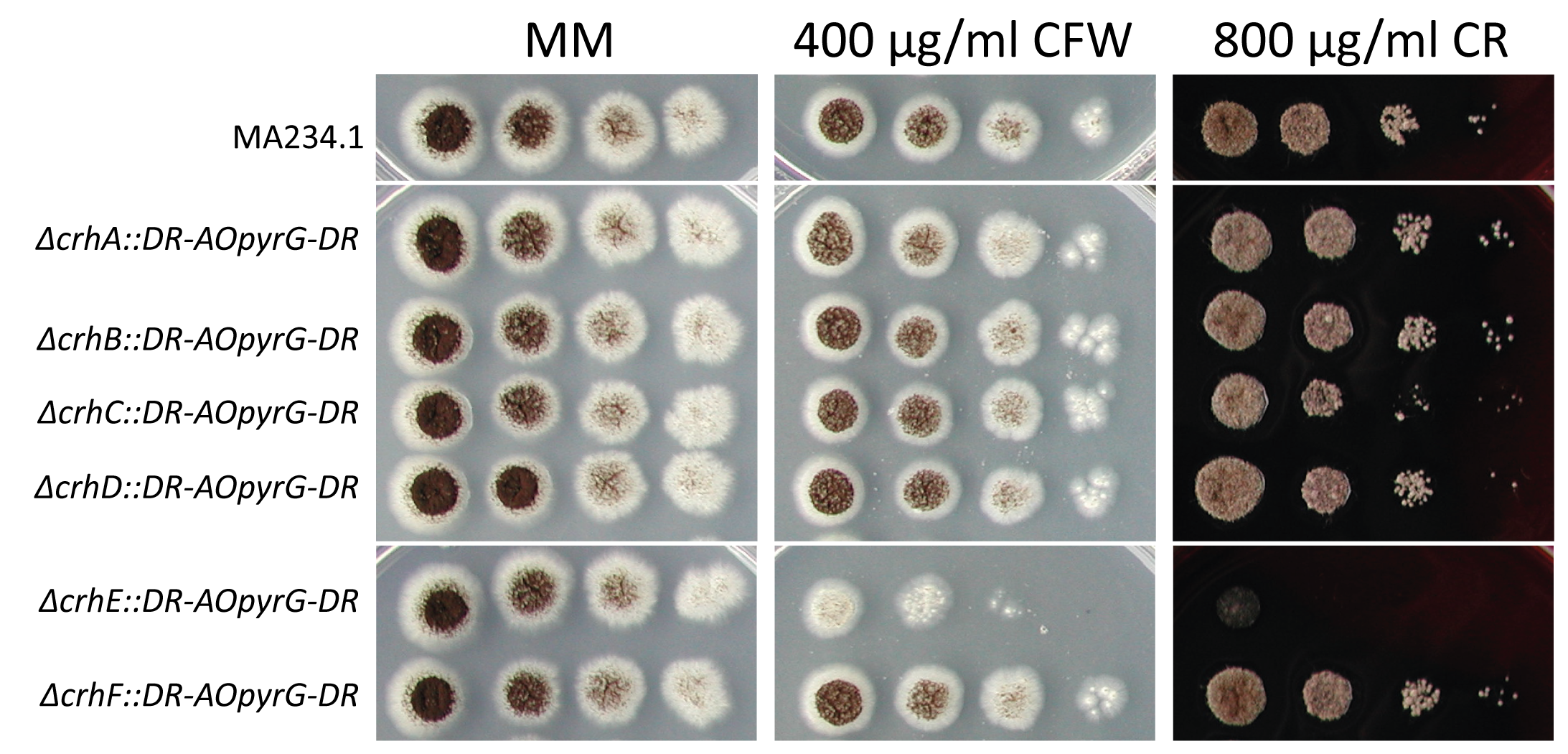

Supplement: Supplementary file 6 — Additional file 6: Figure S5. Growth morphology of single crh knockout strains obtained via replacing the respective crh gene with DR-split marker AOpyrG in MA169.4; ∆crhA-F on MM, MM + 400 µg/mL CFW or MM + 800 µg/mL CR. [file 40694_2019_76_MOESM6_ESM.tif]

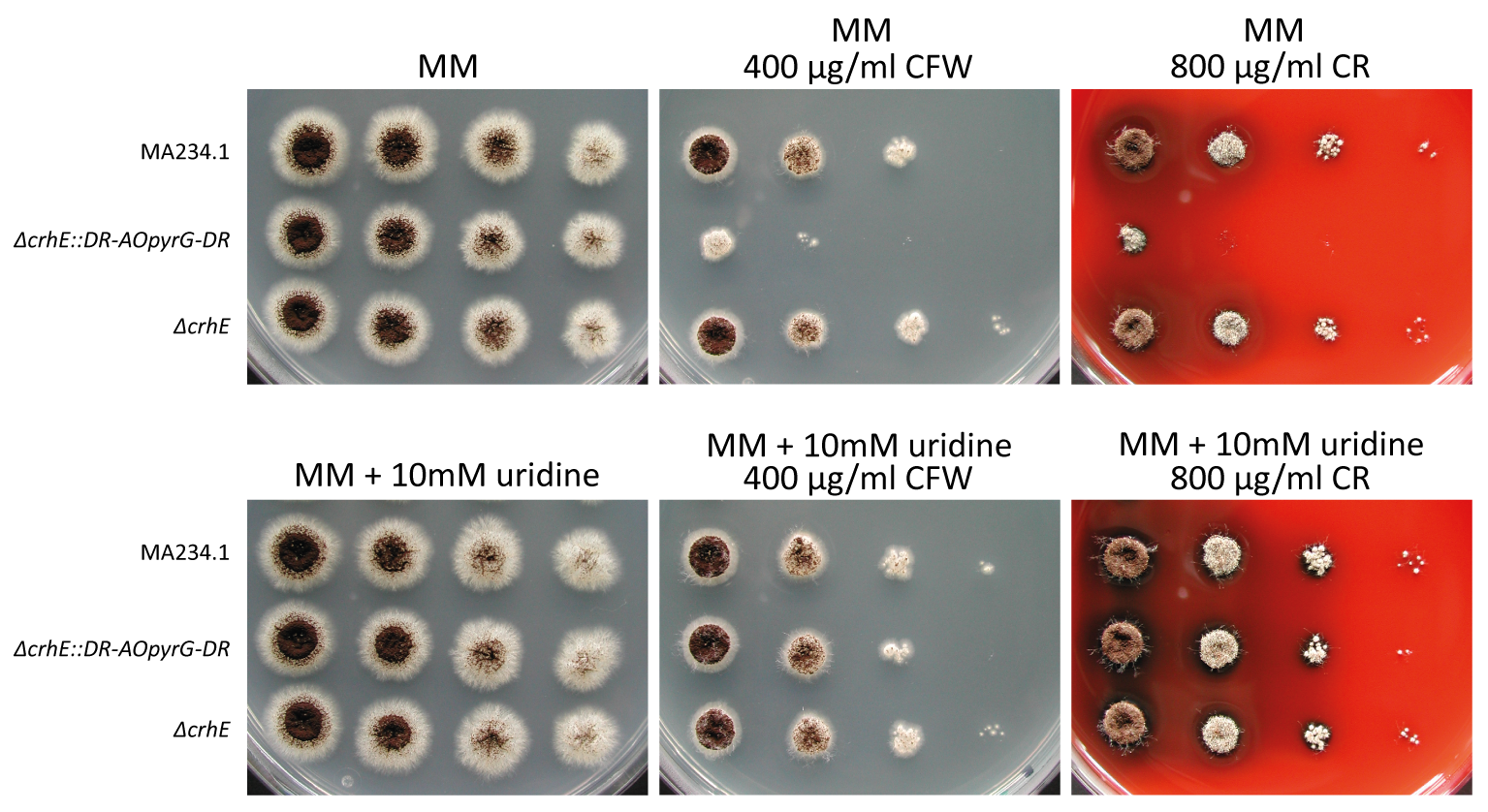

Supplement: Supplementary file 7 — Additional file 7: Figure S6. Growth morphology of MA234.1, ΔcrhE::DR-AOpyrG-DR and ΔcrhE. (A) Strains were grown on MM, MM + 400 µg/mL CFW or MM + 800 µg/mL CR. (B) Additionally, strains were grown on MM + 10 mM uridine, MM + 10 mM uridine + 400 µg/mL CFW)and MM + 10 mM uridine + 800 µg/mL CR. [file 40694_2019_76_MOESM7_ESM.tif]
