# Supplementary material for: Efficient marker free CRISPR/Cas9 genome editing for functional analysis of gene families in filamentous fungi
Source: Fungal Biol Biotechnol. 2019 Sep 21;6:13. doi: 10.1186/s40694-019-0076-7 (PMC6754632; doi:10.1186/s40694-019-0076-7)
Supplement: Supplementary file 4 — Additional file 4: Table S1. All primers used in this study. [file 40694_2019_76_MOESM4_ESM.docx]

**Additional file 4: Table S1.** All primers used in this study.

| **Primer** | **Target** | **Sequence** | **Used for** | **Product size (bp)** | **Notes** |
| --- | --- | --- | --- | --- | --- |
| OTL50 | FW *crhD* HDR check | GGCCCAATGTACCTGATCC | Diagnostic PCR | 4207 | Primes outside of OTL233 to diagnostically PCR the correct insert through HDR |
| OTL51 | RV *crhD* HDR check | GCTCAGCTCTACCCTGAACG | Diagnostic PCR | 4207 | Primes outside of OTL236 to diagnostically PCR the correct insert through HDR |
| OTL56 | FW *crhB* HDR check | CAACCGGGGTTTATTTGTTG | Diagnostic PCR | 4549 | Primes outside of OTL225 to diagnostically PCR the correct insert through HDR |
| OTL57 | RV *crhB* HDR check | TCTTGGCCGACTTCCTAGAC | Diagnostic PCR | 4549 | Primes outside of OTL228 to diagnostically PCR the correct insert through HDR |
| OTL62 | FW *crhF* HDR check | GTTCAGTGGCATCCACCAAG | Diagnostic PCR | 3331 | Primes outside of OTL237 to diagnostically PCR the correct insert through HDR |
| OTL63 | RV *crhF* HDR check | GAATGCATATGATCCGTCCA | Diagnostic PCR | 3331 | Primes outside of OTL240 to diagnostically PCR the correct insert through HDR |
| OTL68 | FW *crhC* HDR check | CGCGCAACTCCTCAATTT | Diagnostic PCR | 3432 | Primes outside of OTL229 to diagnostically PCR the correct insert through HDR |
| OTL69 | RV *crhC* HDR check | TGCAGCAGCATCTCCTTCTA | Diagnostic PCR | 3432 | Primes outside of OTL232 to diagnostically PCR the correct insert through HDR |
| OTL74 | FW *crhE* HDR check | CCCTCAGGTTCAGACTCGAC | Diagnostic PCR | 3461 | Primes outside of OTL221 to diagnostically PCR the correct insert through HDR |
| OTL75 | RV *crhE* HDR check | AAGGGACGTTAGATGCATGG | Diagnostic PCR | 3461 | Primes outside of OTL224 to diagnostically PCR the correct insert through HDR |
| OTL94 | FW *crhA* HDR check | TGTTACAATGGTTCCCCACA | Diagnostic PCR | 4507 | Primes outside of OTL217 to diagnostically PCR the correct insert through HDR |
| OTL95 | RV *crhA* HDR check | CTCAATCAGCCCCTTCAATC | Diagnostic PCR | 4507 | Primes outside of OTL220 to diagnostically PCR the correct insert through HDR |
| OTL100 | FW *crhG* HDR check | TCAACTTCCTGTCTGGGAATG | Diagnostic PCR | 3748 | Primes outside of OTL241 to diagnostically PCR the correct insert through HDR |
| OTL101 | RV *crhG* HDR check | TCATCCTTCATGCTGATCCA | Diagnostic PCR | 3748 | Primes outside of OTL244 to diagnostically PCR the correct insert through HDR |
| pTE1_for | Pro1-promoter sgRNA | CCttaattaaACTCCGCCGAACGTACTG | 5' flank sgRNA expression cassette | 273 | *Pac*I site (orange lower case) + CC overhang for ligation in pFC332. Used i.c.w. a reverse (Rv) sgRNA primer |
| pTE1_rev | Terminator sgRNA | CCttaattaaAAAAGCAAAAAAGGAAGGTACAAAAAAGC | 3' flank sgRNA expression cassette | 142 | *Pac*I site (orange lower case) + CC overhang for ligation in pFC332. Used i.c.w. a forward (Fw) sgRNA primer |
| OTL161 | sgRNA *crhA*_Rv | ggatcattatgggtagctccGACGAGCTTACTCGTTTCG | pRC-target *crhA* | 273 | Reverse complement target site is shown in lower case. Pro1-promoter binding site is indicated in uppercase |
| OTL162 | sgRNA_*crhA*_Fw | ggagctacccataatgatccGTTTTAGAGCTAGAAATAGCAAG | pTarget *crhA* | 142 | Target site in 5' to 3' orientation is shown in lower case. sgRNA binding site is indicated in uppercase |
| OTL163 | sgRNA *crhB*_Rv | tgtgtgagagcaagacctacGACGAGCTTACTCGTTTCG | pRC-target *crhB* | 273 | Reverse complement target site is shown in lower case. Pro1-promoter binding site is indicated in uppercase |
| OTL164 | sgRNA_*crhB*_Fw | gtaggtcttgctctcacacaGTTTTAGAGCTAGAAATAGCAAG | pTarget *crhB* | 142 | Target site in 5' to 3' orientation is shown in lower case. sgRNA binding site is indicated in uppercase |
| OTL167 | sgRNA *crhD*_Rv | cagccaacgcaacagcagtcGACGAGCTTACTCGTTTCG | pRC-target *crhD* | 273 | Reverse complement target site is shown in lower case. Pro1-promoter binding site is indicated in uppercase |
| OTL168 | sgRNA_*crhD*_Fw | gactgctgttgcgttggctgGTTTTAGAGCTAGAAATAGCAAG | pTarget *crhD* | 142 | Target site in 5' to 3' orientation is shown in lower case. sgRNA binding site is indicated in uppercase |
| OTL169 | sgRNA *crhE*_Rv | tctatcacgccaagacgagcGACGAGCTTACTCGTTTCG | pRC-target *crhE* | 273 | Reverse complement target site is shown in lower case. Pro1-promoter binding site is indicated in uppercase |
| OTL170 | sgRNA_*crhE*_Fw | gctcgtcttggcgtgatagaGTTTTAGAGCTAGAAATAGCAAG | pTarget *crhE* | 142 | Target site in 5' to 3' orientation is shown in lower case. sgRNA binding site is indicated in uppercase |
| OTL171 | sgRNA *crhF*_Rv | gtcgaaagatgtgtcgttacGACGAGCTTACTCGTTTCG | pRC-target *crhF* | 273 | Reverse complement target site is shown in lower case. Pro1-promoter binding site is indicated in uppercase |
| OTL172 | sgRNA_*crhF*_Fw | gtaacgacacatctttcgacGTTTTAGAGCTAGAAATAGCAAG | pTarget *crhF* | 142 | Target site in 5' to 3' orientation is shown in lower case. sgRNA binding site is indicated in uppercase |
| OTL173 | sgRNA *crhG*_Rv | attgcaaccccctcaacaccGACGAGCTTACTCGTTTCG | pRC-target *crhG* | 273 | Reverse complement target site is shown in lower case. Pro1-promoter binding site is indicated in uppercase |
| OTL174 | sgRNA_*crhG*_Fw | ggtgttgagggggttgcaatGTTTTAGAGCTAGAAATAGCAAG | pTarget *crhG* | 142 | Target site in 5' to 3' orientation is shown in lower case. sgRNA binding site is indicated in uppercase |
| OTL191 | gRNA_*brnA*_Rev | gcacatattggtaccactccGACGAGCTTACTCGTTTCG | pRC-target *brnA* | 273 | Reverse complement target site is shown in lower case. Pro1-promoter binding site is indicated in uppercase |
| OTL192 | gRNA_*brnA*_Fw | ggagtggtaccaatatgtgcGTTTTAGAGCTAGAAATAGCAAG | pTarget *brnA* | 142 | Target site in 5' to 3' orientation is shown in lower case. sgRNA binding site is indicated in uppercase |
| OTL217 | 5' flank *crhA* KO Rv | GATATCCCGGCGATAATTCA | Knockout repair DNA 5' flank | 950 | Required for the construction of the 5' donor DNA flank for complete removal of *crhA*. Use in combination with OTL218 |
| OTL218 | 5'flank *crhA* KO Fw | GGGAGGACGAAATTTCTGAG | Knockout repair DNA 5' flank | 950 | Required for the construction of the 5' donor DNA flank for complete removal of *crhA*. Use in combination with OTL217 |
| OTL219 | 3' flank *crhA* KO Fw | tgaattatcgccgggatatcGCCAAGCTTGACTGTTTCCT | Knockout repair DNA 3' flank | 825 | Overhang (lower case) matches OTL217 for fusion PCR. Required for the construction of the 3' donor DNA flank for complete removal of *crhA*. Use in combination with OTL220 |
| OTL220 | 3'flank *crhA* KO Rv | CAAAAATCAAGCGGGAAATG | Knockout repair DNA 3' flank | 825 | Required for the construction of the 3' donor DNA flank for complete removal of *crhA*. Use in combination with OTL219 |
| OTL221 | 5' flank *crhE* KO Rv | AATGAACGGTCTGAGCGAGT | Knockout repair DNA 5' flank | 901 | Required for the construction of the 5' donor DNA flank for complete removal of *crhE*. Use in combination with OTL222 |
| OTL222 | 5'flank *crhE* KO Fw | TGACACACATGGTCACCAGA | Knockout repair DNA 5' flank | 901 | Required for the construction of the 5' donor DNA flank for complete removal of *crhE*. Use in combination with OTL221 |
| OTL223 | 3' flank *crhE* KO Fw | actcgctcagaccgttcattGGCTGTGCCATTTGTACTGA | Knockout repair DNA 3' flank | 845 | Overhang (lower case) matches OTL221 for fusion PCR. Required for the construction of the 3' donor DNA flank for complete removal of *crhE*. Use in combination with OTL224 |
| OTL224 | 3'flank *crhE* KO Rv | TGGTATCCAATGCAGTGAGG | Knockout repair DNA 3' flank | 845 | Required for the construction of the 3' donor DNA flank for complete removal of *crhE*. Use in combination with OTL223 |
| OTL225 | 5'flank *crhB* KO Fw | TAACAGATCATCGGGCCAAC | Knockout repair DNA 5' flank | 892 | Required for the construction of the 5' donor DNA flank for complete removal of *crhB*. Use in combination with OTL226 |
| OTL226 | 5'flank *crhB* KO Rv | TTGGTCTGGAGATGTGCAAG | Knockout repair DNA 5' flank | 892 | Required for the construction of the 5' donor DNA flank for complete removal of *crhB*. Use in combination with OTL225 |
| OTL227 | 3'flank *crhB* KO Fw | cttgcacatctccagaccaaGGAGACTCGACAAGCAATCA | Knockout repair DNA 3' flank | 902 | Overhang (lower case) matches OTL226 for fusion PCR. Required for the construction of the 3' donor DNA flank for complete removal of *crhB*. Use in combination with OTL228 |
| OTL228 | 3'flank *crhB* KO Rv | TCCCCCACAGCTACTGAAAC | Knockout repair DNA 3' flank | 902 | Required for the construction of the 3' donor DNA flank for complete removal of *crhB*. Use in combination with OTL227 |
| OTL229 | 5'flank *crhC* KO Fw | GATCTACGCAGGACCGAGAC | Knockout repair DNA 5' flank | 898 | Required for the construction of the 5' donor DNA flank for complete removal of *crhC*. Use in combination with OTL230 |
| OTL230 | 5'flank *crhC* KO Rv | GGGTCCGAACGAGAATGTTA | Knockout repair DNA 5' flank | 898 | Required for the construction of the 5' donor DNA flank for complete removal of *crhC*. Use in combination with OTL229 |
| OTL231 | 3'flank *crhC* KO Fw | taacattctcgttcggacccGCGAGATGCTTGATGTGGTA | Knockout repair DNA 3' flank | 901 | Overhang (lower case) matches OTL230 for fusion PCR. Required for the construction of the 3' donor DNA flank for complete removal of *crhC*. Use in combination with OTL232 |
| OTL232 | 3'flank *crhC* KO Rv | GTCCTCCTCCTCATCACCAA | Knockout repair DNA 3' flank | 901 | Required for the construction of the 3' donor DNA flank for complete removal of *crhC*. Use in combination with OTL231 |
| OTL233 | 5'flank *crhD* KO Fw | GTTGATGACCCTCGGTTTCA | Knockout repair DNA 5' flank | 948 | Required for the construction of the 5' donor DNA flank for complete removal of *crhD*. Use in combination with OTL234 |
| OTL234 | 5'flank *crhD* KO Rv | TCTTCAATGCCAACAACGAG | Knockout repair DNA 5' flank | 948 | Required for the construction of the 5' donor DNA flank for complete removal of *crhD*. Use in combination with OTL233 |
| OTL235 | 3'flank *crhD* KO Fw | ctcgttgttggcattgaagaTCTGGGTCGTTTGGAAACTT | Knockout repair DNA 3' flank | 902 | Overhang (lower case) matches OTL234 for fusion PCR. Required for the construction of the 3' donor DNA flank for complete removal of *crhD*. Use in combination with OTL236 |
| OTL236 | 3'flank *crhD* KO Rv | TCTTGGGCGTCAACTTACCT | Knockout repair DNA 3' flank | 902 | Required for the construction of the 3' donor DNA flank for complete removal of *crhD*. Use in combination with OTL235 |
| OTL237 | 5'flank *crhF* KO Fw | GGTCGATTTGTTTCATCGTG | Knockout repair DNA 5' flank | 901 | Required for the construction of the 5' donor FNA flank for complete removal of *crhF*. Use in combination with OTL238 |
| OTL238 | 5'flank *crhF* KO Rv | CTCCCCGGTAGAGGTTTTGT | Knockout repair DNA 5' flank | 901 | Required for the construction of the 5' donor FNA flank for complete removal of *crhF*. Use in combination with OTL237 |
| OTL239 | 3'flank *crhF* KO Fw | acaaaacctctaccggggagCGAAAACGATGAATCCAACC | Knockout repair DNA 3' flank | 957 | Overhang (lower case) matches OTL238 for fusion PCR. Required for the construction of the 3' donor FNA flank for complete removal of *crhF*. Use in combination with OTL210 |
| OTL240 | 3'flank *crhF* KO Rv | GATGGTAGAAGCAACGATCCA | Knockout repair DNA 3' flank | 957 | Required for the construction of the 3' donor FNA flank for complete removal of *crhF*. Use in combination with OTL239 |
| OTL241 | 5'flank *crhG* KO Fw | GGAAAGTGGCAGATCGACTAA | Knockout repair DNA 5' flank | 952 | Required for the construction of the 5' donor GNA flank for complete removal of *crhG*. Use in combination with OTL242 |
| OTL242 | 5'flank *crhG* KO Rv | CGCGTGGTAGGAGTAATGGT | Knockout repair DNA 5' flank | 952 | Required for the construction of the 5' donor GNA flank for complete removal of *crhG*. Use in combination with OTL241 |
| OTL243 | 3'flank *crhG* KO Fw | accattactcctaccacgcgTTGTGCGGAATGTTTTACGA | Knockout repair DNA 3' flank | 896 | Overhang (lower case) matches OTL242 for fusion PCR. Required for the construction of the 3' donor GNA flank for complete removal of *crhG*. Use in combination with OTL244 |
| OTL244 | 3'flank *crhG* KO Rv | GTGTGAAATGAGGGGGTGAG | Knockout repair DNA 3' flank | 896 | Required for the construction of the 3' donor GNA flank for complete removal of *crhG*. Use in combination with OTL243 |
| OTL247 | sgRNA3 *crhC*_Rv | cgacttgcagcaccgacagcGACGAGCTTACTCGTTTCG | pRC-target *crhC* | 273 | Reverse complement target site is shown in lower case. Pro1-promoter binding site is indicated in uppercase |
| OTL248 | sgRNA3_*crhC*_Fw | gctgtcggtgctgcaagtcgGTTTTAGAGCTAGAAATAGCAAG | pTarget *crhC* | 142 | Target site in 5' to 3' orientation is shown in lower case. sgRNA binding site is indicated in uppercase |
| OTL249 | 5' flank *brnA* KO Fw | TTGAGAACATCGTGGTAGCC | Knockout repair DNA 5' flank | 924 | Required for the construction of the 5' donor DNA flank for complete removal of *brnA*. Use in combination with OTL250 |
| OTL250 | 5' flank *brnA* KO Rv | GCAGACTTTCGGAGCAATTC | Knockout repair DNA 5' flank | 924 | Required for the construction of the 5' donor DNA flank for complete removal of *brnA*. Use in combination with OTL249 |
| OTL251 | 3' flank *brnA* KO Fw | gaattgctccgaaagtctgcCCATATTGAGTGGCACGTTG | Knockout repair DNA 3' flank | 932 | Required for the construction of the 3' donor DNA flank for complete removal of *brnA*. Use in combination with OTL252 |
| OTL252 | 3' flank *brnA* KO Rv | ATTGGGTGGTGGGACAATTA | Knockout repair DNA 3' flank | 932 | Required for the construction of the 3' donor DNA flank for complete removal of *brnA*. Use in combination with OTL251 |
| OTL380 | *brnA* HDR check Fw | TTGGGGTGAGAGTTTGCTTT | Diagnostic PCR | 2411 | Use to check for seamles integration of repair DNA after CRISPR transformation. Primes 314bp upstream of OTL249 |
| OTL381 | *brnA* HDR check Rv | GAGGGAGGGGTTTTATCTCG | Diagnostic PCR | 2411 | Use to check for seamles integration of repair DNA after CRISPR transformation. Primes 233bp downstream of OTL252 |
| OTL487 | Rv Promoter tRNA_Pro1 (gRNA) | GACGAGCTTACTCGTTTCG | 5' flank sgRNA expression cassette | 253 | Use in combination with pTE1_for to create the 5' flank of the gRNA construct. Does not include a (R&C) target overhang. This primer was designed to amplify the 5' flank of the gRNA (tRNA Pro1 promoter) for cloning and storage in pJET1.2 |
| OTL488 | Fw trRNA-terminator (gRNA) | GTTTTAGAGCTAGAAATAGCAAG | 3' flank sgRNA expression cassette | 122 | Use in combination with pTE1_rev to create the 3' flank of the gRNA construct. Does not include a target overhang. This primer was designed to amplify the 3' flank of the gRNA (trRNA and terminator) for cloning and storage in pJET1.2 |
